# Communicating breastfeeding benefits or formula‐feeding risks? The underlying process explaining the framing effect on infant‐feeding attitudes and intentions

**DOI:** 10.1111/aphw.70105

**Published:** 2026-02-02

**Authors:** Margherita Guidetti, Giulia Scaglioni, Nicoletta Cavazza

**Affiliations:** ^1^ Department of Communication and Economics University of Modena and Reggio Emilia Reggio Emilia Italy

**Keywords:** affective reactions, breastfeeding promotion, gain–loss framing, health communication, information acceptance, self‐efficacy

## Abstract

A preregistered experimental study tested the effects of message framing on breastfeeding and formula‐feeding attitudes and intentions. It also examined whether affective reaction and information acceptance mediated these effects, and whether self‐efficacy and perceived behavioral control (PBC) moderated them. Participants (282 pregnant women) were randomly assigned to a gain frame condition (benefits of breastfeeding), a loss frame condition (risks of not breastfeeding), or a control condition. Results showed two opposite indirect effects: the loss frame elicited negative affect, which lowered information acceptance; and conversely, the gain frame induced positive affect, thus increasing acceptance. These affective and cognitive responses differentially affected breastfeeding and formula‐feeding attitudes and intentions, with the loss frame indirectly worsening the former (95% CI [−.24, −.08]) and improving the latter (95% CI [.03, .11]), while the gain frame worsened formula‐feeding attitudes and intentions (95% CI [−.03, −.01]) and improved those related to breastfeeding (95% CI [.01, .08]). Additionally, low levels of breastfeeding self‐efficacy and PBC amplified the negative effects of the loss‐framed message and suppressed the positive effects of the gain‐framed message. These findings highlight the affective and cognitive mechanisms through which risk‐based language can have unintended, counterproductive effects. Breastfeeding promotion should emphasize benefits rather than risks and empower women's self‐efficacy.

## INTRODUCTION

The short‐ and long‐term benefits of breastfeeding for both mothers and infants are widely established and universally acknowledged (Victora et al., 2016). In children, including those from high‐income countries, breastfeeding reduces mortality due to infectious diseases (Sankar et al., 2015), hospitalization for preventable diseases such as gastroenteritis, respiratory disease, and otitis media (Bowatte et al., 2015; Horta, 2013), lowers the risk of childhood diabetes, overweight, and obesity (Horta et al., 2015a), and contributes to higher intelligence quotient (Horta et al., 2015b). For women, breastfeeding is associated with a decreased risk of breast and ovarian cancer, as well as diabetes (Chowdhury et al., 2015). Many of these benefits are dose‐dependent, with the greatest advantages resulting from exclusive breastfeeding (with no added food or fluids) for approximately 6 months, followed by continued breastfeeding alongside complementary foods (Kramer & Kakuma, 2012).

Accordingly, the World Health Organization, UNICEF (2002), many national governments, and professional associations such as the American Academy of Pediatrics (Meek et al., 2022) recommend exclusive breastfeeding for the first 6 months of life, and continued breastfeeding with appropriate complementary foods up to 2 years of age or beyond (depending on maternal and infant preference). If these guidelines were universally followed, 823,000 deaths in children under 5 and 20,000 deaths from breast cancer could be prevented each year (Victora et al., 2016). From an economic and social perspective, beyond the notable expenditure on formula milk for families, and the high environmental cost of its production, packaging, and transportation, not breastfeeding has a huge economic cost due to children's lower cognitive development and higher morbidity (Rollins et al., 2016).

Notwithstanding the improvements observed in the last few years (WHO, 2024), the variability in breastfeeding rates (North et al., 2022) and the current low prevalence of exclusive breastfeeding up to 6 months (i.e., about 25% in both Europe and the United States; CDC Breastfeeding Report Card, 2022; Theurich et al., 2019) require a deeper understanding of the most effective communication strategies to promote breastfeeding and discourage formula‐feeding. The present study aimed to test the differential effectiveness of communicating either the advantages of breastfeeding or the disadvantages of formula‐feeding, while also considering the underlying affective and cognitive processes, as well as individual differences in breastfeeding self‐efficacy and perceived behavioral control (PBC). As for the practical implications, understanding which communication frame works better can guide health campaigns. This applied purpose also sheds light on the broader issue of the loss–gain framing effect in health communication and the mechanisms driving it.

### Breastfeeding is normal

Since Wiessinger's (1996) paper entitled “Watch your language,” breastfeeding advocates have been criticizing the slogan “breast is best” as inappropriate and misleading. The fact that formula manufacturers often use this same sentence should raise suspicions about its ability to promote breastfeeding. According to Wiessinger (1996), if breastfeeding is presented as the optimal, perfect, ideal, and special choice, parents may be tempted to settle for the “normal” option (i.e., formula) because perfection implies extra commitment, is unrealistic, and unnecessary. Talking about breastfeeding advantages does “reinforce bottle feeding yet again as the accepted, acceptable norm” (Wiessinger, 1996, p. 1). Indeed, health policies usually treat the biological norm as the baseline, the reference point for determining whether a deviation is a gain or a loss (e.g., smoking is presented as harmful rather than quitting smoking as beneficial). Breastfeeding is the biological norm, the default against which the alternative of formula‐feeding should be compared. If breastfeeding is normal, then formula‐feeding should be presented as “deficient, incomplete, and inferior” (Wiessinger, 1996, p. 1).

Wiessinger's appeal produced a progressive shift from benefit to risk language as a widespread communicative practice (e.g., Wallace & Ofuokwu, 2024). However, aside from the moral problems the loss frame raises (Woollard, 2018), a few empirical studies have shown that it is not more effective than the gain frame.

### Framing effect and health behavior

The framing effect is the phenomenon by which the same consequences presented as gain or loss produce different preferences: people tend to be risk‐averse when facing potential gains and risk‐seeking when facing potential losses. This is explained by prospect theory (Tversky & Kahneman, 1981), which predicts that the displeasure of a loss is at least twice the pleasure of an equivalent gain. Therefore, setting breastfeeding as the reference point and framing formula‐feeding as a loss should be a more effective strategy than the reverse.

However, Rothman and Salovey (1997) hypothesized that, in health communication, the direction of the framing effect varies according to the behavior considered: whereas loss‐framed messages would be more effective in promoting detection behaviors (i.e., risk‐seeking behavior), gain‐framed messages would be more effective in promoting prevention behaviors (i.e., risk‐averse behavior). Many studies (e.g., Garcia‐Retamero & Cokely, 2011; Rivers et al., 2005; Rothman et al., 1999) and a recent meta‐analysis (Nabi et al., 2020) confirmed Rothman and Salovey's hypothesis across a range of health behaviors (but see also Gallagher & Updegraff, 2012; O'Keefe & Jensen, 2007, 2009).

Exclusive breastfeeding for up to 6 months can be conceived as a prevention behavior. Thus, gain‐framed messages might be more effective than loss‐framed messages in promoting breastfeeding. However, the few studies testing the framing effect on breastfeeding attitudes and intentions reported mixed findings. In line with Rothman and Salovey's hypothesis (1997), Hussein et al. (2014), involving a sample of Indonesian pregnant women, found that a gain‐framed message (only when delivered by a credible source) produced the most favorable attitudes and intentions toward breastfeeding. In contrast, Bakker and Van Acke (2014), in a study conducted with nonpregnant Dutch women, found no evidence of a framing effect. However, their sample was small, the disadvantages of breastfeeding were also included in the messages, and only attitude was assessed. In addition, this assessment was repeated after each piece of information was provided. Nonetheless, similar findings were reported by Wallace and Taylor, who exposed American university students (both women and men; Wallace & Taylor, 2011) and pregnant women (Wallace & Taylor, 2016) to either a benefits‐ or risks‐framed message, finding no differences in breastfeeding intentions. More recently, the same authors conducted a longitudinal study comparing actual infant‐feeding behaviors, again failing to find a framing effect in either direction (Wallace & Taylor, 2021).

However, prior research has some limitations that our study has addressed. First, previous studies did not explore the underlying mechanisms that might produce differential indirect effects for benefits and risks communication. Moreover, individual differences potentially moderating these effects were not considered. Finally, because most studies compared gain and loss frames without including a control condition,^1^ it remains unclear whether both frames positively affected the outcomes.

### The underlying process: The mediating role of affect and information acceptance and the moderating role of self‐efficacy

The emotions as frames model (Nabi, 2003, 2007) argues that different messages induce different emotions, motivating the accessibility of and search for specific information. This model suggests that positive and negative emotions may mediate the effects of positive and negative frames. Although evidence for the total effects of gain–loss framing on persuasion remains limited, a recent meta‐analysis provided support for this hypothesis (Nabi et al., 2020). However, beyond the role of emotional intensity for both gain‐ and loss‐framed communications, several questions remain unanswered. The following appears especially relevant for our purpose: “Are there implications for how the produced emotion influences message engagement in other ways, for example, processing depth, perceived message effectiveness, and the like? A closer consideration of the psychological state induced by gain‐loss framing would be helpful in building theory around its process of influence via emotional intensity” (Nabi et al., 2020, p. 1123).

For instance, a gain‐framed message about reducing salt intake fostered the corresponding intention through positive affect, which in turn increased information acceptance and attitude. In contrast, the effect of the loss‐framed message on intention was mediated only by negative affect, without passing through information acceptance and attitude (Van't Riet et al., 2010). Still, perhaps also depending on the health behavior considered, negative affect can reduce information acceptance. Indeed, in Wallace and Taylor's studies (2011, 2016), participants exposed to a message about the risks of formula found it less trustworthy, accurate, and helpful than those exposed to a message about the benefits of breastfeeding. These authors, however, did not assess participants' affective reactions, nor did they test a mediational model linking information acceptance to infant‐feeding intentions.

In addition to the type of behavior investigated, the intensity of negative emotions elicited by loss‐framed messages may also depend on individual characteristics such as self‐efficacy and PBC. In fact, such messages can be considered fear appeals (Van't Riet et al., 2010), which may prompt message denial and derogation when the recipient feels unable to ward off the threat by adopting the recommended action (Kessels et al., 2014; Ruiter et al., 2001). The extended parallel process model (Witte, 1992) and the protection motivation theory (Rogers, 1983) propose that the impact of threat on intention is moderated by efficacy perception. Both efficacy (Witte, 1992) and coping (Rogers, 1983) appraisals include response efficacy (i.e., confidence that the recommended behavior will reduce the risk) and self‐efficacy (i.e., confidence in one's ability to perform the recommended behavior). Many empirical findings confirmed the hypothesized moderating role of efficacy perception: A meta‐analysis showed an improvement in effect size of *d* = +.68 on intentions for risk‐based communication in which both self‐ and response efficacy were heightened (Sheeran et al., 2014). Since self‐efficacy and PBC are crucial predictors of breastfeeding (e.g., Lau et al., 2018) and can reduce maternal stress (Law et al., 2019), we focused on these constructs as moderators of both the loss‐framed and the gain‐framed message effects.

We thus expected that the loss‐framed message would elicit less positive and more negative affective reactions, thereby lowering information acceptance compared to the control condition. However, this negative effect should be neutralized or even reversed when recipients show high levels of breastfeeding self‐efficacy and PBC. These opposing effects could be an additional reason why the gain frame works better than the loss frame for prevention behaviors, which usually require stronger and more prolonged commitment than detection behaviors. Conversely, the gain‐framed message would induce more positive and less negative affect than the control message, thus fostering information acceptance; this should apply to all participants, yet particularly to those characterized by high levels of self‐efficacy and PBC. Indeed, confidence in one's own ability to perform the beneficial behavior should strengthen the positive reaction to the benefits appraisal.

### Doing and not doing

Finally, most investigations on the framing effect compared a message about the gain of performing a behavior with a message about the loss of not performing the same behavior, yet focusing on performing the target behavior as the dependent variable. However, some studies indicate the importance of examining cognitions and intentions related to both performing and not performing a target action, since both affect actual behavior (e.g., Richetin et al., 2011). This seems particularly relevant when the studied behavior—that is, breastfeeding—has one main conceptual alternative, namely, formula‐feeding (when the mother's or a donor's expressed milk is unavailable). Indeed, the goals and motivations underlying these behaviors are not simply opposite but different. For instance, a study showed that one of the main reasons underlying the breastfeeding choice was the health benefits for the baby, whereas one of the main reasons for formula‐feeding was mothers' perception of their partners' attitude (Arora et al., 2000).

Therefore, based on a matching effect, messages contending the gain of performing and the loss of not performing a behavior may have differential impacts on cognitions and intentions concerning both performing and not performing the target action, with stronger effects on the corresponding outcomes.

### The present study

This study aimed to compare the effect of a gain‐framed message, a loss‐framed message, and a control condition in affecting pregnant women's breastfeeding and formula‐feeding attitudes and intentions, also testing the mediating role of affective reaction and information acceptance, and the moderating role of self‐efficacy and PBC. While prior research on risks and benefits language focused either on framing effect in health communications (Bakker & Van Acke, 2014; Hussein et al., 2014) or on Wissinger's appeal (Wallace & Taylor, 2011, 2016, 2021), our study brought together these two separate literatures. By including a control condition and focusing on the affective and cognitive processes underlying the gain–loss framing effect, we addressed the limitations of previous research on breastfeeding promotion. Furthermore, to the best of our knowledge, no prior study has examined individual differences that potentially moderate the effects of benefit and risk communication, or assessed these effects on both breastfeeding and formula‐feeding attitudes and intentions. Finally, since our model can be applied to (and tested with) other prevention or detection health behaviors, it offers a theoretical contribution to understanding the complex mechanisms at play when people receive gain‐ or loss‐framed messages.

**FIGURE 1:**
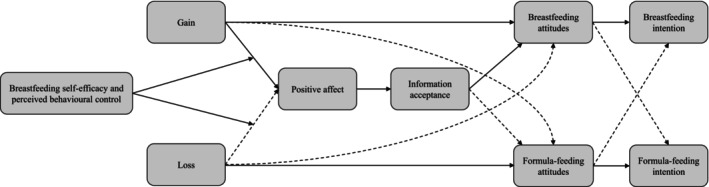
The hypothesized moderated mediation model. Dashed lines indicate negative paths.

We hypothesized the moderated mediation model depicted in Figure 1. The effects of gain‐framed and especially loss‐framed messages should depend on participants' level of breastfeeding self‐efficacy and PBC. The gain‐framed message should improve participants' breastfeeding attitudes and intentions (Hp1a) and worsen their formula‐feeding attitudes and intentions (Hp1b) by increasing positive and decreasing negative affect and thus increasing information acceptance (Hp1c). These effects should emerge particularly for those scoring high on self‐efficacy and PBC, but to a lesser extent also for those scoring low (Hp1d). As this message concerns performing the target behavior, its effects could be stronger for breastfeeding attitudes and intentions (performing) than for formula‐feeding attitudes and intentions (not performing the behavior; Hp1e).

Conversely, the effect of the loss‐framed message should more strongly depend on participants' level of breastfeeding self‐efficacy and PBC: For those scoring high, the loss‐framed message could improve participants' breastfeeding attitudes and intentions (Hp2a) and worsen participants' formula‐feeding attitudes and intentions (Hp2b) by reducing negative and increasing positive affect and thus fostering information acceptance (Hp2c). In contrast, for those scoring low on self‐efficacy and PBC, the loss‐framed message should have the backlash effect of worsening breastfeeding (Hp3a) and improving formula‐feeding attitudes and intentions (Hp3b) by increasing negative and reducing positive affect and thus hindering information acceptance (Hp3c). These effects could also be stronger for formula‐feeding (not performing) than for breastfeeding (performing the behavior; Hp3d).

We also expected the effects on intentions to be partially mediated by attitudes (Hp4a). Finally, as the loss‐framed message should have opposite effects as a function of participants' level of self‐efficacy and PBC, we anticipated that the gain‐framed message would be more effective for the whole sample (Hp4b).

Although this study had autonomous research aims, it used and pretested materials for a larger project approved by the Ethical Committee of the Area Vasta Emilia Nord. Method and hypotheses were preregistered before data collection on Open Science Framework: https://doi.org/10.17605/OSF.IO/KFWQD Our preregistration included the study design, planned sample size, inclusion/exclusion criteria, and planned primary analyses. The materials, data, and syntax of analyses supporting our findings are openly available at https://osf.io/jntqy/overview?view_only=bd872fc9abcd49bdbf7e0a7d9e8e9753. We report all manipulations, measures, and exclusions.

## METHOD

### Participants

Based on an a priori power analysis conducted with G*Power (Faul et al., 2007), we set the goal of recruiting at least 252 participants, a sufficient sample to detect a medium effect size of *f* = 0.25, with α = .05 and power = .95 for a mixed model ANOVA with three groups and two measurements. We recruited English‐native‐speaking^2^ pregnant women on Prolific, which remunerated their participation with £0.90.

**FIGURE 2:**
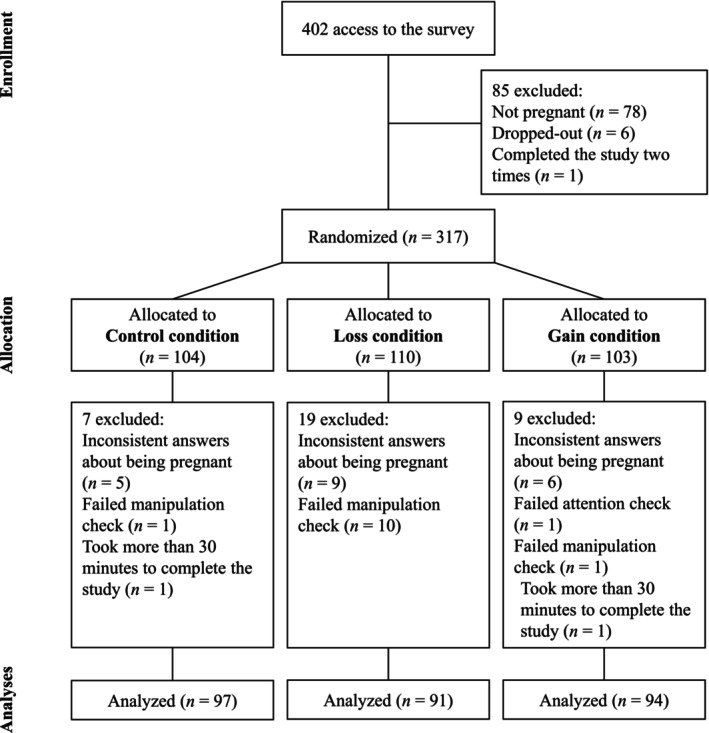
Participants flow chart.

The survey was accessed 402 times (Figure 2). Participants were not allowed to complete the study if they were not pregnant. Some users attempted to take the survey a second time, stating they were pregnant. Because of the inconsistency of their answers, these cases were excluded from the analyses. Another Prolific user completed the study twice, and only the first response was retained. Additional exclusion criteria included taking an excessively long time to complete the survey (i.e., more than 30 min) and failing either the attention or the manipulation checks (see below in *Procedure*). After accounting for exclusions and dropouts (see Figure 2), the final sample consisted of 282 participants.^3^

Participants' ages ranged from 19 to 50 years (*M* = 30.98, *SD* = 4.87). In terms of education, 64.5% of participants had a bachelor's degree or higher, while 35.5% had a lower level of education. Most participants were from the United Kingdom (*n* = 167), followed by the United States (*n* = 79), South Africa (*n* = 17), Australia (*n* = 8), Ireland (*n* = 6), Canada (*n* = 3), and Israel (*n* = 1). All participants were at least 4 months pregnant (*M* = 22 weeks pregnant), and 54.4% of participants had at least one previous child. Among these, 26% reported no prior breastfeeding, and 50% reported no prior exclusive breastfeeding (overall, 60% of the participants had never breastfed, and 73% had never exclusively breastfed).

### Procedure

Participants provided written informed consent before completing an anonymous questionnaire on Qualtrics. The initial questions validated participants' pregnancy status (e.g., whether they were pregnant and their gestational week). Next, we reported a definition of *exclusive breastfeeding* and measured participants' sense of self‐efficacy and PBC over exclusive breastfeeding up to 6 months. Subsequently, the survey platform randomly assigned participants to one of three conditions: a gain frame condition highlighting the benefits of breastfeeding, a loss frame condition emphasizing the risks of formula‐feeding, or a control condition presenting neutral facts about animals. After reading the assigned messages, respondents were asked about the content of what they read (manipulation check^4^), the affective reactions induced by the messages, and their level of information acceptance.

The following section of the questionnaire assessed the dependent variables: intention to exclusively breastfeed for 6 months, affective and cognitive attitudes toward exclusive breastfeeding, intention to formula‐feed, and affective and cognitive attitudes toward formula‐feeding. In the final section, we asked respondents whether they had other children, the number and age of those children, past infant‐feeding practices, and sociodemographic information (sex, age, education, country of residence, and size of the municipality of residence).

The survey included two attention checks. The first, placed before the experimental messages, asked participants if they swim across the Atlantic Ocean to get to work every day. The second, placed before the dependent variables, instructed participants to select “Green” as the response to the question: “What is your favorite color?”

### Experimental materials

Participants randomly assigned to the “gain frame condition” read a list of information introduced by the sentence: “Scientific research has consistently shown that breastfeeding has many advantages for both babies and mothers.” In the “loss frame condition,” the introduction read: “Scientific research has consistently shown that not breastfeeding (i.e., feeding babies formula) has many disadvantages for both babies and mothers,” and the subsequent content mirrored that of the gain condition. These introductory sentences were included because source credibility had a crucial role in Hussein et al.'s study (2014). Depending on the experimental condition, participants then read either the advantages of breastfeeding or the disadvantages of formula‐feeding for the infant (e.g., favors/hinders the physiological development of the mouth) and for the mother (e.g., helps/hinders the loss of weight accumulated during pregnancy). The information provided in the experimental conditions was adapted from materials by the Italian Ministry of Health (2019), Victora et al. (2016), and Krol and Grossman (2018). In the control condition, participants read factual information about animals (e.g., flamingos are pink because of the shrimps they eat).

Each condition included 23 slides with 18 pieces of information and counted around 230 words (see shared materials on OSF). All the slides from the same condition appeared on a single survey page and remained visible for at least 60 s (participants could not proceed to the next section before this time had elapsed).

### Measures

The items assessing breastfeeding self‐efficacy and PBC, as well as breastfeeding and formula‐feeding attitudes and intentions, were adapted from previous studies on breastfeeding framed in the theory of planned behavior (Grano et al., 2023; Lawton et al., 2012; Richetin et al., 2011).

### Moderators and mediators

#### Breastfeeding self‐efficacy and PBC

Participants completed two items assessing self‐efficacy (“For me, exclusively breastfeeding my baby up to the age of 6 months will be very easy,” “I'm sure I'll be able to exclusively breastfeed my baby up to 6 months if I want to”) and two items measuring PBC (“Even if I decide to exclusively breastfeed my baby up to 6 months old, something could happen that will force me to stop,” “Exclusively breastfeeding my baby for 6 months is totally up to me”). Items were answered on a 5‐point Likert scale. An explorative factorial analysis (EFA) revealed a single factor explaining 50.27% of the variance. Three out of four factor loadings varied from .73 to .84. However, the factor loading of “Exclusively breastfeeding my baby for 6 months is totally up to me” was quite low (.31) and noticeably worsened the Cronbach's α from .73 (without the item) to .66 (with the item). We thus computed a single score by averaging the other three items. A higher score corresponds to greater levels of breastfeeding self‐efficacy and PBC (“self‐efficacy” from here onwards).

#### Affective reaction to the messages and information acceptance

Van't Riet et al. (2010, Study 2) used a set of eight adjectives to capture participants' affective reactions to health‐related messages (about salt intake). From their list, we selected four items and replaced *afraid* with *worried*, as we considered it more appropriate to the specific topic and messages of our study. Hence, we asked respondents how much the information they had just read made them feel *happy*, *optimistic*, *worried*, and *uncomfortable*. The four items were presented in randomized order and rated on a 5‐point Likert scale from 1 (*Not at all*) to 5 (*Very much*). An EFA showed that the four items loaded on a single factor explaining 62% of the variance. Thus, a mean score was computed (α = .79) after reverse‐coding the negative items, so that a higher score corresponds to more positive affect.

To measure information acceptance, participants in the gain and loss frame conditions had to evaluate the messages as *convincing*, *useful*, *unrealistic* (reverse‐coded), and *exaggerated* (reverse‐coded). These adjectives were presented in randomized order and rated on the same 5‐point scale used for the affect items. As an EFA revealed a single factor explaining 78% of variance, a mean score was calculated (α = .91) so that a higher score corresponds to greater information acceptance.

Since the information provided in the control condition was unrelated to breastfeeding and formula‐feeding and thus could not affect infant‐feeding attitudes and intentions, we did not assess information acceptance in this case. Instead, we assigned all participants in the control condition a neutral score of 3 (i.e., the scale midpoint). Affect, however, was measured across all conditions as they might influence infant‐feeding attitudes and intentions even when elicited by unrelated content.

### Dependent variables

#### Breastfeeding and formula‐feeding attitudes

The affective component of attitudes toward breastfeeding and formula‐feeding was measured through eight semantic differential items (four for each behavior): “For me, exclusively breastfeeding my baby up to 6 months/formula‐feeding would be …” pleasant–unpleasant (reverse‐coded), relaxing–tiring (reverse‐coded), inconvenient−convenient, and embarrassing–a reason to be proud. Similarly, the cognitive component of attitudes toward breastfeeding and formula‐feeding was measured through the following differential items: healthy–unhealthy (reverse‐coded), safe–risky (reverse‐coded), disadvantageous–advantageous, and stupid–wise. The differential items were presented in randomized order. Participants answered on a 5‐point scale. An EFA revealed a two‐factor structure explaining 71.48% of the variance for breastfeeding attitude and a single factor across affective and cognitive components explaining 72.84% of the variance for formula‐feeding attitude. However, items concerning breastfeeding were not clearly distinct as affective and cognitive, and the reliability of the full scale was very good. Therefore, also for consistency between the two measures, a single mean score was computed for both so that higher scores indicate a more positive attitude toward breastfeeding (α = .88) and formula‐feeding (α = .90).

#### Breastfeeding and formula‐feeding intentions

Mothers' intention to breastfeed was measured through 3 items: “Do you intend to exclusively breastfeed your baby up to 6 months old?” (1, *Definitely do not*; 5, *Definitely do*), “How strong is your intention to exclusively breastfeed your baby up to 6 months old?” (1, *Very strong*; 5, *Very weak*, reverse coded), “How likely is it that you will exclusively breastfeed your baby up to 6 months old?” (1, *Very unlikely*; 5, *Very likely*). Conversely, formula‐feeding intention was measured through the following items: “Do you intend to formula‐feed your baby?” (1, *Definitely do not*; 5, *Definitely do*), “How strong is your intention to formula‐feed your baby?” (1, *Very strong*; 5, *Very weak*, reverse coded), “How likely is it that you will formula‐feed your baby?” (1, *Very unlikely*; 5, *Very likely*). A mean score was computed for both breastfeeding (α = .94) and formula‐feeding intentions (α = .94).

### Data analyses

First, we ran two 2 × 3 mixed model analyses of variance (ANOVAs), one on breastfeeding and formula‐feeding attitudes and the other on breastfeeding and formula‐feeding intentions as within‐participants factors, with experimental condition as the between‐participants factor. We expected a main effect of condition, with the gain‐framed message being more effective (i.e., increasing the delta between breastfeeding and formula‐feeding ratings) than both the control and loss conditions (Hp4b). We also expected an interaction effect, with the gain‐framed message being more effective for breastfeeding attitudes and intentions (performing the behavior) than for formula‐feeding attitudes and intentions (not performing the behavior; Hp1e), and the loss‐framed message being more effective for formula‐feeding attitudes and intentions (not performing the behavior) than for breastfeeding attitudes and intentions (performing the behavior; Hp3d).

Next, we tested half of the model at a time, using Process, the SPSS macro provided by Hayes (2018). We customized Model 92 so that it would not test the moderating effect of self‐efficacy on the link between information acceptance and attitudes and between attitudes and intentions. The main outcome of the first model was breastfeeding intention. We entered experimental condition as the independent variable (two dummy variables for gain and loss, with control as the reference), affective reaction, and information acceptance as sequential mediators, followed by both breastfeeding attitudes and formula‐feeding attitudes as parallel mediators, and breastfeeding self‐efficacy as the moderator (Hp1a, Hp1c, Hp1d, Hp2b, Hp2c, Hp3b, Hp3c, and Hp4b). The second model was run on formula‐feeding intentions (Hp1b, Hp1c, Hp1d, Hp2a, Hp2c, Hp3a, Hp3c, and Hp4b). Hp1e and Hp3d were also tested by comparing the confidence intervals of the indirect effects yielded from these models.

Finally, we tested the full model (Figure 1) by running a path analysis using AMOS. This allowed us to simultaneously consider both dependent variables and their (expected) correlation, as well as to estimate the total effects.

## RESULTS

Descriptive statistics and correlations among measures are displayed in Table 1.

**TABLE 1:** Descriptive statistics and correlations.

	*M* (*SD*)				Correlations					
	Full sample	Control	Loss	Gain	2	3	4	5	6	7
1. Self‐efficacy	3.06 (.94)	2.99 (1.05)	3.04 (.88)	3.15 (.88)	.23	.28	.62	−.51	.71	−.70
2. Emotions	3.45 (1.09)	3.76^b^ (.59)	2.40^a^ (.89)	4.16^c^ (.89)		.60	.32	−.23	.26	−.22
3. Information acceptance	3.33 (1.02)	3.00^a^ (.00)	2.81^a^ (1.18)	4.17^b^ (.83)			.47	−.35	.39	−.36
4. Breastfeeding attitude	3.92 (.79)	3.78^a^ (.84)	3.85^a^ (.78)	4.12^b^ (.70)				−.48	.82	−.76
5. Formula‐feeding attitude	3.26 (.92)	3.27 (.91)	3.31 (.88)	3.20 (.96)					−.53	.65
6. Breastfeeding intention	3.69 (1.28)	3.62 (1.38)	3.55 (1.29)	3.89 (1.17)						−.85
7. Formula‐feeding intention	2.46 (1.18)	2.59 (1.24)	2.49 (1.22)	2.30 (1.06)						

*Note*: Means with different superscripts are significantly different.

### Total effects

Both mixed model ANOVAs only yielded a significant effect of the within‐participants factor: breastfeeding attitudes were more positive than formula‐feeding attitudes, *F*(1, 279) = 56.69, *p* < .001, *η*_*p*_^2^ = .17, *f* = .45, 95% CI [.32, .57], and breastfeeding intentions were greater than formula‐feeding intentions, *F*(1, 279) = 75.94, *p* < .001, *η*_*p*_^2^ = .21, *f* = .52, 95% CI [.39, .64]. Although the interaction between attitudes and the experimental condition was not statistically significant, *F*(2, 279) = 2.35, *p* = .097, a multivariate analysis of variance run on both attitudes and intentions showed a significant effect of the experimental condition only on breastfeeding attitudes, *F*(2, 279) = 5.09, *p* = .007, *η*_*p*_^2^ = .04, *f* = .17, 95% CI [.05, .30]. Bonferroni pairwise comparisons showed that the gain‐framed message resulted in more positive breastfeeding attitudes than both loss‐framed (*p* = .009) and control conditions (*p* = .048). Therefore, Hp4b and Hp3d were not supported, and Hp1e was only partially supported, as far as gain and breastfeeding attitudes are concerned.

### Test of the moderated mediation model

**FIGURE 3:**
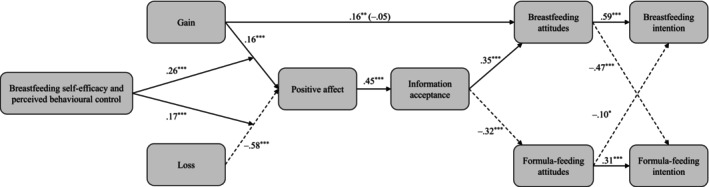
Results of the moderated mediation model. Standardized regression coefficients are reported. Dashed lines indicate negative paths.

Although the lack of significant total effects on the breast‐ and formula‐feeding intentions, the further analyses run with Process and Amos confirmed the expected model (Figure 3), explaining a high portion of variance for both breastfeeding, *R* ^2^ = .75, *F*(9, 272) = 89.25, *p* < .001, *f* ^2^ = 3.00, and formula‐feeding intentions, *R* ^2^ = .72, *F*(9, 272) = 76.51, *p* < .001, *f* ^2^ = 2.57. The path analysis showed a perfect fit to the data: *χ* ^2^(5) = 2.09, *p* = .836, TLI = 1.02, CFI = 1.00, RMSEA = .00 (90% CI: .00–.05).

### The moderating role of self‐efficacy

**FIGURE 4:**
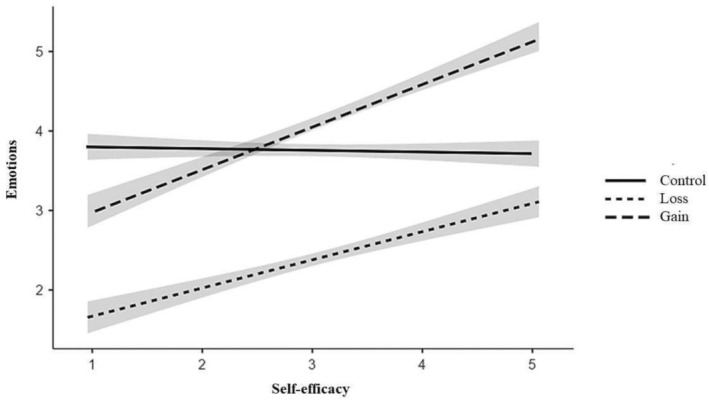
Experimental conditions' effects on emotions as a function of participants' self‐efficacy level.

The Process models yielded the expected interactions (with some differences) between both experimental conditions and self‐efficacy on affect (see Figure 4): The gain‐framed message failed to induce positive affect when participants' self‐efficacy was low, simple effect = −.17, *SE* = .15, *t* = −1.09, *p* = .277, 95% CI [−.46, .13]. In contrast, it did have a positive effect when self‐efficacy was moderate, simple effect = .36, *SE* = .11, *t* = 3.36, *p* = .001, 95% CI [.15, .57], and high, simple effect = .88, *SE* = .15, *t* = 5.96, *p* < .001, 95% CI [.59, 1.17]; *b* = .56, *SE* = .11, *t* = 4.96, *p* < .001, 95% CI [.34, .78]. In a mirrorlike way, at low levels of self‐efficacy, the loss‐framed message significantly decreased positive affect, simple effect = −1.71, *SE* = .15, *t* = −11.55, *p* < .001, 95% CI [−2.00, −1.42], but this negative impact diminished at moderate levels of self‐efficacy, simple effect = −1.36, *SE* = .11, *t* = −12.65, *p* < .001, 95% CI [−1.57, −1.15], and at high levels of self‐efficacy, simple effect = −1.00, *SE* = .15, *t* = −6.51, *p* < .001, 95% CI [−1.31, −.70]; *b* = .37, *SE* = .11, *t* = 3.32, *p* < .001, 95% CI [.15, .60], though retaining significance and not changing sign as anticipated (Hp2c). Figure 4 also illustrates that the gain frame had a more positive impact on affect than the loss frame across all levels of self‐efficacy, providing initial support for Hp4b.

### The conditional indirect effects of loss‐ and gain‐framed messages

Table 2 displays the conditional indirect effects of experimental conditions on breastfeeding and formula‐feeding intentions. The hypothesized positive indirect effect of the gain frame on breastfeeding intentions—operating through affective reaction, information acceptance, and both breastfeeding and formula‐feeding attitudes― was not significant at low levels of self‐efficacy but emerged as significant and positive when self‐efficacy was moderate and high. A similar pattern was found for the negative indirect effect of the gain‐framed message on formula‐feeding intentions (operating through affect, information acceptance, and both breastfeeding and formula‐feeding attitudes): It failed to reach significance at low levels of self‐efficacy, whereas it was significant at moderate and high levels. Hypotheses Hp1a, Hp1b, and Hp1c were thus supported. Hypothesis Hp1d was only partially supported because the gain messages failed to indirectly increase breastfeeding and decrease formula‐feeding intentions, compared to the control condition, for women reporting low self‐efficacy, while we expected weaker—but still present―indirect effects also for them.

**TABLE 2:** Indirect effects of experimental conditions (vs. control condition) on breastfeeding and formula‐feeding intentions.

	Loss frame				Gain frame			
Condition → emotions → info acceptance → breastfeeding attittudes → breastfeeding intention								
Self‐efficacy	Effect	*SE*	95% LLCI	95% ULCI	Effect	*SE*	95% LLCI	95% ULCI
Low	−.19	.05	−.299	−.105	−.02	.02	−.065	.020
Moderate	−.15	.04	−.237	−.084	.04	.02	.014	.076
High	−.11	.03	−.189	−.056	.10	.03	.052	.162
Index of mod. med.	.04	.02	.014	.080	.06	.02	.030	.109

Condition → emotions → info acceptance → formula‐feeding attitudes → breastfeeding intention								
Self‐efficacy	Effect	*SE*	95% LLCI	95% ULCI	Effect	*SE*	95% LLCI	95% ULCI
Low	−.03	.02	−.064	−.004	.00	.00	−.012	.003
Moderate	−.02	.01	−.050	−.003	.01	.00	.001	.015
High	−.02	.01	−.038	−.002	.02	.01	.002	.033
Index of mod. med.	.01	.00	.001	.017	.01	.01	.001	.022

Condition → emotions → info acceptance → breastfeeding attitudes → formula‐feeding intention								
Self‐efficacy	Effect	SE	95% LLCI	95% ULCI	Effect	SE	95% LLCI	95% ULCI
Low	.14	.04	.074	.220	.01	.02	−.015	.047
Moderate	.11	.03	.059	.173	−.03	.01	−.055	−.010
High	.08	.02	.040	.135	−.07	.02	−.119	−.037
Index of mod. med.	−.03	.01	−.060	−.100	−.05	.02	−.079	−.020

Condition → emotions → info acceptance → formula‐feeding attitudes → formula‐feeding intention								
Self‐efficacy	Effect	SE	95% LLCI	95% ULCI	Effect	SE	95% LLCI	95% ULCI
Low	.08	.03	.043	.142	.01	.01	−.009	.031
Moderate	.07	.02	.034	.111	−.02	.01	−.035	−.006
High	.05	.02	.024	.084	−.04	.02	−.077	−.021
Index of mod. med.	−.02	.01	−.039	−.006	−.03	.01	−.053	−.011

On the other hand, the indirect effects of the loss frame were significant across the full sample, negative for breastfeeding intentions and positive for formula‐feeding intentions. This contradicts hypotheses Hp2a, Hp2b, and Hp2c, which predicted that the loss frame should have resulted in stronger breastfeeding and weaker formula‐feeding intentions for women scoring high on breastfeeding self‐efficacy. Instead, these findings support hypotheses Hp3a, Hp3b, and Hp3c for the whole sample. However, the hypothesized moderating role of self‐efficacy was confirmed as both the indirect effects of the loss frame (negative on breastfeeding and positive on formula‐feeding) were stronger for participants scoring low on the self‐efficacy measure and weakened at increasing levels of self‐efficacy, though retaining significance. Detailed results breaking down these indirect effects are reported in the S1 Information (Section 1).

### Comparing the indirect effects

The indexes of moderated mediation at moderate levels of self‐efficacy are equivalent to the unconditional indirect effects. We therefore used them to compare the relative influence of the experimental conditions on breast‐ and formula‐feeding attitudes and intentions, in order to test a potential matching effect (Hp1e and Hp3d). The absolute values of their confidence intervals partially overlapped (see Table 2), indicating that the indirect effect of the gain‐framed message on breastfeeding intentions was not significantly stronger than its indirect effect on formula‐feeding intentions. Hence, Hp1e was not supported. In addition, the indirect effect of the loss‐framed message on formula‐feeding intentions was significantly weaker than its indirect effect on breastfeeding intentions, thus contradicting Hp3d.

As predicted (Hp4a), the observed effects on both intentions were mediated by attitudes. Notably, all these indirect effects on breastfeeding and formula‐feeding intentions were stronger when passing through breastfeeding attitudes than formula‐feeding attitudes (see Table 2). This suggests that beliefs about breastfeeding are more influential than those on formula‐feeding, not only on breastfeeding intentions but also on formula‐feeding intentions. Consistently, Figure 3 shows that both breastfeeding and formula‐feeding intentions were more strongly predicted by breastfeeding than formula‐feeding attitudes. Hypothesis Hp4b was supported: across the full sample, the gain‐framed message was more effective than the loss‐framed one. However, this was not due to the expected opposite effects of the loss frame but to its negative effects on all participants, including high self‐efficacy women.

## DISCUSSION

The discourse surrounding breastfeeding promotion often deals with the need to present breastfeeding as the biological norm rather than the best option. From this perspective, it seems more appropriate to highlight the risks of not breastfeeding rather than the benefits of breastfeeding (Wiessinger, 1996). We conceptually agree with this reasoning, but our experimental study suggests that risk‐based communication may even be counterproductive: All participants reacted negatively to the loss‐framed message, and this backlash effect was particularly pronounced in women less confident about their breastfeeding abilities. Therefore, these results confirmed that gain‐framed communication is more effective than loss‐framed communication in promoting a preventive health behavior such as breastfeeding (Rothman & Salovey, 1997). Importantly, gain‐framed messages also appear to be more aligned with parents' own preferences than loss‐framed messages (Brown et al., 2020), an aspect that should not be overlooked if we are to move beyond a paternalistic model of health communication and embrace an approach grounded in citizen engagement and participatory practices.

In line with prior studies involving Western samples (Wallace & Taylor, 2011, 2016), we did not find a significant total effect of our manipulation on breastfeeding intentions. However, as Hayes (2018, p. 117) notes, “the total effect simply is not a good way of thinking about X's effect on Y.” Indeed, by testing a moderated mediation model, we detected both main and conditional indirect effects of gain‐ and loss‐framed messages.^5^ While we expected self‐efficacy to moderate the impact of the loss‐framed message more strongly than the gain‐framed one, the pattern we observed was the opposite. A comparison of standardized regression coefficients (see Figure 3) revealed that the loss‐framed message had a stronger main effect on affective reaction than the gain frame. However, the impact of the gain‐framed message was more strongly moderated by participants' self‐efficacy than the impact of the loss frame. In other words, the loss frame had negative effects for all participants, though more negative for those low in self‐efficacy, whereas the gain‐framed message had positive effects only among women with moderate to high self‐efficacy.

The present findings shed light on the underlying process involved in the framing effect of messages promoting breastfeeding: receiving information about the risks of not breastfeeding induced negative affect, which in turn hindered information acceptance. Although the information was rejected, this did not result in a null effect on infant‐feeding attitudes and intentions. In contrast, dismissing risk‐based information about formula‐feeding led to more negative attitudes and intentions toward breastfeeding and more positive ones toward formula‐feeding compared to the baseline control condition. In other words, the opportunity to inform pregnant women about the difference between breast‐ and formula‐feeding was not only missed but even backfired in a counterproductive reaction. Unexpectedly, this was also damaging for high self‐efficacy women, though less detrimental than for low self‐efficacy women.

This finding is only partially consistent with the well‐documented role of self‐efficacy as a moderator of fear appeals' effectiveness (Sheeran et al., 2014). The partial inconsistency lies in the fact that the negative effects of the loss‐framed message did not turn into positive effects at high levels of self‐efficacy. This could depend on the specific nature of the behavior under consideration. From a philosophical perspective, Woollard (2018) argued that framing infant‐feeding decisions in terms of harm and danger carries strong moral implications, contributing to mothers' guilt and shame, with negative consequences on their well‐being. In addition, even if breastfeeding is the biological norm, it cannot be treated as the default for comparison, like not smoking or not adhering to a screening: indeed, “breastfeeding is doing something,” and something that requires “the mother's body and agency” (Woollard, 2018, p. 758). Therefore, the risk language may trigger negative reactions that are so strong that not even confident individuals can reinterpret them as a positive challenge.

In contrast, emphasizing the benefits of breastfeeding generally improved participants' affective reaction to the messages and thus information acceptance. This information could then correspondingly affect breast‐ and formula‐feeding attitudes and intentions. However, participants' level of self‐efficacy moderated their affective reaction to the gain‐framed messages: Low self‐efficacy women exhibited neutral affect, not differing from the scale midpoint, yet less positive than those in the control group who received irrelevant information about animals. Consistently, the indirect effect of the gain‐framed message was not significant for low self‐efficacy participants. In other words, any message is ineffective for women who lack confidence in their ability to breastfeed.

Since mothers who had not breastfed their previous children and those who had not breastfed exclusively showed the lowest levels of self‐efficacy (with the breastfeeding and exclusively breastfeeding mothers placed at highest levels and the childless women falling in between, *F*(2, 275) = 29.02, *p* < .001, *η* ^2^ = .17 and *F*(2, 275) = 75.05, *p* < .001, *η* ^2^ = .35), this result aligns with a recent qualitative study suggesting that even the “breast is best” narrative can trigger negative feelings of guilt and shame in mothers feeding their babies formula, including those who also breastfeed (Scott & Bute, 2025). This points to the need to improve women's breastfeeding self‐efficacy and PBC, with a particular focus on mothers who may have had a difficult past breastfeeding experience. Indeed, these constructs are not only key predictors of breastfeeding intentions and actual behavior (Golnam et al., 2025; Lau et al., 2018), but our study showed they are also crucial factors in modulating the effectiveness of breastfeeding promotion. Fostering women's self‐efficacy means providing accurate information about breastfeeding physiology and how to facilitate the activation of the innate skills of both mother and baby, while also raising awareness about potential difficulties and strategies to overcome them (Sousa et al., 2025).

Our study also aimed to explore whether presenting the gain of performing versus the loss of not performing a target behavior would have different effects on attitudes and intentions related to performing or not performing that behavior. The results showed that the indirect effects observed on breastfeeding and formula‐feeding attitudes and intentions were specular. While the effects of information acceptance on the two attitude measures mirrored each other, breastfeeding attitudes were more influential than formula‐feeding attitudes on both breast‐ and formula‐feeding intentions.

Inevitably, this study has a few limitations. First, as often happens in sociopsychological experiments, pregnant women recruited as participants are a convenience sample. However, we at least managed to include women of different sociocultural contexts. Some may consider this heterogeneity as a drawback, since different countries have different norms about breast‐ and formula‐feeding. However, an independent sample *t*‐test revealed no significant differences in the mean scores of any assessed variables between UK and US participants (i.e., the two most numerous subsamples), *p*s > .070. In addition, the main analyses were rerun separately for these two groups. Despite the lower statistical power due to the smaller sample sizes, the results were substantially the same (see Supporting Information, Sections 2–3, for details). Although further research is needed, this suggests that our findings may be quite replicable across contexts and, therefore, generalizable.

Second, actual behavior was not assessed, so we cannot know whether the observed effects on breast‐ and formula‐feeding intentions extended to real infant‐feeding practices. However, we do not consider this a major limitation because the goal of educational interventions like those we tested is to improve actual breastfeeding rates by enhancing breastfeeding intentions. Though we are aware of the intention‐behavior gap for health behaviors in general (Sheeran & Webb, 2016) and breastfeeding in particular (Guo et al., 2016), we can assume that intentions would mediate any potential effect of these interventions on behavior.

Notwithstanding these limitations, the present results have the strength of integrating different strands of literature, whereas prior research on risks and benefits language has focused either on the framing effect in health communications (Bakker & Van Acke, 2014; Hussein et al., 2014) or on Wissinger's appeal (Wallace & Taylor, 2011, 2016, 2021). In addition, including a control condition allowed us to compare gain and loss framing effects against a no‐intervention baseline. Other original elements adding value to this work include the identification of the affective and cognitive processes driving loss‐ and gain‐framed communication effects, the estimation of these effects on both breastfeeding and formula‐feeding attitudes and intentions, and the uncovering of the moderating role of self‐efficacy.

## CONCLUSIONS

The present study illustrated the negative indirect effects of a loss‐framed message and the positive indirect effect of a gain‐framed message on pregnant women's infant‐feeding intentions and, more importantly, the underlying process explaining why benefit‐ and risk‐based communication strategies produce these different effects. In addition, we showed that breastfeeding self‐efficacy and PBC have a key role in moderating the effectiveness of breastfeeding promotion interventions.

From a theoretical perspective, to the best of our knowledge, this is the first study to consider the affective and cognitive processes underlying the framing effect in health communications together with an important individual characteristic such as self‐efficacy. Therefore, this work contributes to the literature on the emotions as frames model (Nabi, 2003, 2007), the extended parallel process model (Witte, 1992), and the protection motivation theory (Rogers, 1983), suggesting that negative affect aroused from a loss frame is not always effective in motivating the recommended behavior, even among high self‐efficacy recipients. Further studies could apply our model to different prevention and detection behaviors, also distinguishing them according to their perceived difficulties. For instance, self‐efficacy may be less relevant for behavior perceived as easy to perform. Therefore, our model could help understand why the loss frame is more effective for detection behaviors, which are likely considered easier than prevention ones.

Breastfeeding is a particular kind of prevention behavior: It requires the mother's commitment and social support, so it is not the typical default of doing nothing; it “cannot be characterized as a neutral state of non‐interference” (Woollard, 2018, p. 758). In addition, breastfeeding involves another person the mother is supposed to care for. This is why it is a morally loaded behavior, and a “morally neutral reframing of breastfeeding as the norm” is very difficult to put into practice (Woollard, 2018, p. 757). We attempted to attain this in our experimental messages by neutrally presenting advantages and disadvantages. However, the loss‐framed message still triggered strong affective reactions.

### Implications

Though it is conceptually convincing that the biological norm of breastfeeding should be taken as the reference point, as claimed by Wiessinger (1996), this risk‐based language is problematic when directed at mothers, not only from a moral perspective (Woollard, 2018), and not only because it does not work as intended (Wallace & Taylor, 2011, 2016, 2021), but also because it can be counterproductive. Even among healthcare providers, a Japanese study showed that nurses and midwives were more inclined to accept and appreciate professional education about the benefits of breastfeeding rather than the risks of formula‐feeding (Toda et al., 2023).

We argue that the negative indirect effects of the loss frame cannot be resolved as Wiessinger (2021) proposes, that is by separating breastfeeding promoters (“e.g., researchers, journal editors, national and international policymakers, and news media,” p. 464) who impersonally communicate the “bad news” that formula‐feeding is harmful from protectors and supporters engaged in personal interactions and using a more neutral language. In our experimental messages, the source of information was indicated as “scientific research” and the issue was not negative judgments about breastfeeding advocates but the affective and cognitive mechanisms inducing a backlash effect from loss‐framed communication. Wiessinger (1996) wrote:

Are you the best possible parents? Is your home life ideal? Do you provide optimal meals? Of course not. Those are admirable goals, not minimum standards. Let us rephrase. Is your parenting inadequate? Is your home life subnormal? Do you provide deficient meals? Now it hurts. You may not expect to be far *above* normal, but you certainly do not want to be *below* normal. (p. 1).

What was once believed to be the source of risk language's effectiveness—the hurt it provokes—turned out to be the very reason for its counterproductive impact. Notably, Mikels et al. (2021) reported similar findings in the context of physical activity promotion: Participants in the loss frame condition were less inclined to exercise and less likely to enroll in an exercise program than those in the gain frame condition precisely because the risk‐framed message elicited weaker positive emotions.

Breastfeeding should be presented as normal, not as a moral ideal. Communicating its benefits in a scientifically grounded and emotionally balanced way may be the key to preventing the negative emotions that make persuasive messages backfire.

## CONFLICT OF INTEREST STATEMENT

The authors report there are no competing interests to declare.

## ETHICS STATEMENT

The “Comitato Etico dell'Area Vasta Emilia Nord” approved our materials (approval: AOU0015527/23) on May 17, 2023.

## Supporting information


**Data S1.** Supporting Information.

## Data Availability

The materials, data, and syntax of analyses supporting our findings are openly available at https://osf.io/jntqy.

## ENDNOTES

^1^
Wallace and Taylor's study (2021) was the only exception, and they did not find significant differences between experimental conditions, nor with the control condition.

^2^
This choice derives from practical reasons: Pregnant women are a particular population and, by recruiting them on Prolific, bringing together participants from different countries allowed us to have a sufficiently large sample.

^3^
No participants showed insufficient effort responding. Since eliminating participants with a too‐large Cook's distance (i.e., larger than 4/N) did not change the results, we decided to keep them to avoid power reduction.

^4^
Specifically, participants were asked to indicate whether the information they had just read described the benefits of breastfeeding, the risks of not breastfeeding (i.e., feeding babies formula), the benefits of healthy eating and exercise during pregnancy, the risks of unhealthy eating and a sedentary lifestyle during pregnancy, or some curious discoveries about animals (random order).

^5^
The lack of total effects was due, in most cases, to indirect and direct effects having different signs.
