# Supplementary material for: Communicating breastfeeding benefits or formula‐feeding risks? The underlying process explaining the framing effect on infant‐feeding attitudes and intentions
Source: Appl Psychol Health Well Being. 2026 Feb 2;18(1):e70105. doi: 10.1111/aphw.70105 (PMC12865253; doi:10.1111/aphw.70105)
Supplement: Supplementary file 1 — Data S1. Supporting Information. [file APHW-18-0-s001.docx]

# Supplemental materials

## More detailed findings

The gain-framed message had a main total effect on info acceptance (β = .53, *p* < .001, 95% CI [.45, .60]) partially mediated by emotions (indirect effect β = .07, *p* < .001, 95% CI [.03, .12]), and on breastfeeding attitudes (β = .16, *p* = .004, 95% CI [.05, .26]) fully mediated by emotions and information acceptance (indirect effect β = .21, *p* < .001, 95% CI [.13, .28]). It also had a conditional total effect on information acceptance (β = .25, *p* < .001, 95% CI [.16, .34]) partially mediated by emotions (indirect effect β = .12, *p* < .001, 95% CI [.06, .18]): the gain frame condition improved information acceptance at increasing levels of self-efficacy (from *M* = 3.66 to *M* = 4.59). In contrast, the loss-framed message did not show any main total effects on mediators or dependent variables, but a conditional total effect on information acceptance (β = .21, *p* = .001, 95% CI [.09, .32]): the effect was negative at low levels of self-efficacy (*b* = –.59, *SE* = .15, *p* < .001, 95% CI [–.89, –.28]) whereas it was non-significant at moderate and high levels, *p* > .105. This conditional total effect was partially mediated by emotions (indirect effect β = .08, *p* = .001, 95% CI [.03, .13]). Emotions had significant total effects on both breastfeeding (β = .28, *p* < .001, 95% CI [.13, .42]) and formula-feeding attitudes (β = −.21, *p* = .001, 95% CI [–.35, –.08]), fully mediated by information acceptance (indirect effects β = .16, *p* < .001, 95% CI [.09, .23], and β = −.15, *p* < .001, 95% CI [–.22, –.07], respectively). Information acceptance showed significant total effects on both breastfeeding (β = .29, *p* < .001, 95% CI [.16, .41]) and formula-feeding intentions (β = −.24, *p* = .001, 95% CI [–.37, –.11]), fully mediated by both attitudes (indirect effect β = .24, *p* < .001,95% CI [.15, .33], and β = −.27, *p* < .001, 95% CI [–.36, –.17]).

## Main analyses rerun for the UK subsample (*N* = 167)

| **Test of Between-Participants Effects** | | | | | | | |
| --- | --- | --- | --- | --- | --- | --- | --- |
| Source | DV | Type III Sum of Squares | *df* | Mean square | *F* | *p* | Partial eta squared |
| Corrected Model | Self-efficacy and PBC | 2.976^a^ | 2 | 1.488 | 1.753 | .177 | .021 |
|  | Emotions associated with messages | 94.763^b^ | 2 | 47.381 | 76.948 | .000 | .484 |
|  | Information acceptance | 66.384^c^ | 2 | 33.192 | 55.235 | .000 | .402 |
|  | Brst_Att | 3.851^d^ | 2 | 1.925 | 3.112 | .047 | .037 |
|  | Bott_Att | 1.131^e^ | 2 | .566 | .641 | .528 | .008 |
|  | Intention to breastfeed | 4.973^f^ | 2 | 2.486 | 1.455 | .236 | .017 |
|  | Intention to feed formula | 3.049^g^ | 2 | 1.524 | 1.037 | .357 | .012 |
| Intercept | Self-efficacy and PBC | 1497.254 | 1 | 1497.254 | 1763.454 | .000 | .915 |
|  | Emotions associated with messages | 1878.709 | 1 | 1878.709 | 3051.065 | .000 | .949 |
|  | Information acceptance | 1747.744 | 1 | 1747.744 | 2908.396 | .000 | .947 |
|  | Brst_Att | 2527.072 | 1 | 2527.072 | 4084.782 | .000 | .961 |
|  | Bott_Att | 1866.347 | 1 | 1866.347 | 2116.485 | .000 | .928 |
|  | Intention to breastfeed | 2121.854 | 1 | 2121.854 | 1241.880 | .000 | .883 |
|  | Intention to feed formula | 1028.487 | 1 | 1028.487 | 699.634 | .000 | .810 |
| ExpCond | Self-efficacy and PBC | 2.976 | 2 | 1.488 | 1.753 | .177 | .021 |
|  | Emotions associated with messages | 94.763 | 2 | 47.381 | 76.948 | .000 | .484 |
|  | Information acceptance | 66.384 | 2 | 33.192 | 55.235 | .000 | .402 |
|  | Brst_Att | 3.851 | 2 | 1.925 | 3.112 | .047 | .037 |
|  | Bott_Att | 1.131 | 2 | .566 | .641 | .528 | .008 |
|  | Intention to breastfeed | 4.973 | 2 | 2.486 | 1.455 | .236 | .017 |
|  | Intention to feed formula | 3.049 | 2 | 1.524 | 1.037 | .357 | .012 |
| Error | Self-efficacy and PBC | 139.244 | 164 | .849 |  |  |  |
|  | Emotions associated with messages | 100.984 | 164 | .616 |  |  |  |
|  | Information acceptance | 98.553 | 164 | .601 |  |  |  |
|  | Brst_Att | 101.459 | 164 | .619 |  |  |  |
|  | Bott_Att | 144.618 | 164 | .882 |  |  |  |
|  | Intention to breastfeed | 280.207 | 164 | 1.709 |  |  |  |
|  | Intention to feed formula | 241.086 | 164 | 1.470 |  |  |  |
| Total | Self-efficacy and PBC | 1641.222 | 167 |  |  |  |  |
|  | Emotions associated with messages | 2094.326 | 167 |  |  |  |  |
|  | Information acceptance | 1927.250 | 167 |  |  |  |  |
|  | Brst_Att | 2635.992 | 167 |  |  |  |  |
|  | Bott_Att | 2011.199 | 167 |  |  |  |  |
|  | Intention to breastfeed | 2412.222 | 167 |  |  |  |  |
|  | Intention to feed formula | 1272.111 | 167 |  |  |  |  |
| Corrected Total | Self-efficacy and PBC | 142.220 | 166 |  |  |  |  |
|  | Emotions associated with messages | 195.747 | 166 |  |  |  |  |
|  | Information acceptance | 164.937 | 166 |  |  |  |  |
|  | Brst_Att | 105.310 | 166 |  |  |  |  |
|  | Bott_Att | 145.749 | 166 |  |  |  |  |
|  | Intention to breastfeed | 285.180 | 166 |  |  |  |  |
|  | Intention to feed formula | 244.134 | 166 |  |  |  |  |
| a. *R*^2^ = .021 (Adjusted *R*^2^ = .009) | | | | | | | |
| b.  *R*^2^ = .484 (Adjusted *R*^2^ = .478) | | | | | | | |
| c.  *R*^2^ = .402 (Adjusted *R*^2^ = .395) | | | | | | | |
| d.  *R*^2^ = .037 (Adjusted *R*^2^ = .025) | | | | | | | |
| e.  *R*^2^ = .008 (Adjusted *R*^2^ = -.004) | | | | | | | |
| f.  *R*^2^ = .017 (Adjusted *R*^2^ = .005) | | | | | | | |
| g.  *R*^2^ = .012 (Adjusted *R*^2^ = .000) | | | | | | | |

| **Descriptives** | | | | |
| --- | --- | --- | --- | --- |
|  | Experimental condition | *M* | *SD* | *N* |
| Self-efficacy and PBC | control | 2.8333 | 1.07309 | 56 |
|  | loss | 2.9938 | .82541 | 54 |
|  | gain | 3.1579 | .84305 | 57 |
|  | Totale | 2.9960 | .92560 | 167 |
| Emotions associated with messages | control | 3.6905 | .62335 | 56 |
|  | loss | 2.3056 | .81360 | 54 |
|  | gain | 4.0687 | .89172 | 57 |
|  | Totale | 3.3718 | 1.08591 | 167 |
| Information acceptance | control | 3.0000 | .00000 | 56 |
|  | loss | 2.6111 | 1.05023 | 54 |
|  | gain | 4.0965 | .84615 | 57 |
|  | Totale | 3.2485 | .99679 | 167 |
| Brst_Att | control | 3.7350 | .89264 | 56 |
|  | loss | 3.8433 | .75444 | 54 |
|  | gain | 4.0947 | .70037 | 57 |
|  | Totale | 3.8928 | .79649 | 167 |
| Bott_Att | control | 3.2712 | .92318 | 56 |
|  | loss | 3.4600 | .89050 | 54 |
|  | gain | 3.3004 | .99745 | 57 |
|  | Totale | 3.3422 | .93702 | 167 |
| Intention to breastfeed | control | 3.5000 | 1.41564 | 56 |
|  | loss | 3.3951 | 1.25572 | 54 |
|  | gain | 3.8012 | 1.24221 | 57 |
|  | Totale | 3.5689 | 1.31071 | 167 |
| Intention to feed formula | control | 2.6369 | 1.31238 | 56 |
|  | loss | 2.5000 | 1.19002 | 54 |
|  | gain | 2.3099 | 1.12838 | 57 |
|  | Totale | 2.4810 | 1.21272 | 167 |

| **Pairwise Comparisons** | | | | | | | |
| --- | --- | --- | --- | --- | --- | --- | --- |
| DV | (I) Experimental condition | (J) Experimental condition | Mean Difference (I-J) | *SE* | *p* ^a^ | 95% CI ^a^ | |
|  |  |  |  |  |  | LL | UL |
| Self-efficacy and PBC | control | loss | -.160 | .176 | 1.000 | -.586 | .265 |
|  |  | gain | -.325 | .173 | .189 | -.744 | .095 |
|  | loss | control | .160 | .176 | 1.000 | -.265 | .586 |
|  |  | gain | -.164 | .175 | 1.000 | -.587 | .259 |
|  | gain | control | .325 | .173 | .189 | -.095 | .744 |
|  |  | loss | .164 | .175 | 1.000 | -.259 | .587 |
| Emotions associated with messages | control | loss | 1.385^*^ | .150 | .000 | 1.023 | 1.747 |
|  |  | gain | -.378^*^ | .148 | .034 | -.735 | -.021 |
|  | loss | control | -1.385^*^ | .150 | .000 | -1.747 | -1.023 |
|  |  | gain | -1.763^*^ | .149 | .000 | -2.124 | -1.403 |
|  | gain | control | .378^*^ | .148 | .034 | .021 | .735 |
|  |  | loss | 1.763^*^ | .149 | .000 | 1.403 | 2.124 |
| Information acceptance | control | loss | .389^*^ | .148 | .028 | .031 | .747 |
|  |  | gain | -1.096^*^ | .146 | .000 | -1.449 | -.744 |
|  | loss | control | -.389^*^ | .148 | .028 | -.747 | -.031 |
|  |  | gain | -1.485^*^ | .147 | .000 | -1.841 | -1.129 |
|  | gain | control | 1.096^*^ | .146 | .000 | .744 | 1.449 |
|  |  | loss | 1.485^*^ | .147 | .000 | 1.129 | 1.841 |
| Brst_Att | control | loss | -.108 | .150 | 1.000 | -.471 | .255 |
|  |  | gain | -.360^*^ | .148 | .048 | -.718 | -.002 |
|  | loss | control | .108 | .150 | 1.000 | -.255 | .471 |
|  |  | gain | -.251 | .149 | .283 | -.613 | .110 |
|  | gain | control | .360^*^ | .148 | .048 | .002 | .718 |
|  |  | loss | .251 | .149 | .283 | -.110 | .613 |
| Bott_Att | control | loss | -.189 | .179 | .880 | -.622 | .244 |
|  |  | gain | -.029 | .177 | 1.000 | -.457 | .398 |
|  | loss | control | .189 | .179 | .880 | -.244 | .622 |
|  |  | gain | .160 | .178 | 1.000 | -.272 | .591 |
|  | gain | control | .029 | .177 | 1.000 | -.398 | .457 |
|  |  | loss | -.160 | .178 | 1.000 | -.591 | .272 |
| Intention to breastfeed | control | loss | .105 | .249 | 1.000 | -.498 | .708 |
|  |  | gain | -.301 | .246 | .667 | -.896 | .294 |
|  | loss | control | -.105 | .249 | 1.000 | -.708 | .498 |
|  |  | gain | -.406 | .248 | .311 | -1.007 | .194 |
|  | gain | control | .301 | .246 | .667 | -.294 | .896 |
|  |  | loss | .406 | .248 | .311 | -.194 | 1.007 |
| Intention to feed formula | control | loss | .137 | .231 | 1.000 | -.422 | .696 |
|  |  | gain | .327 | .228 | .461 | -.225 | .879 |
|  | loss | control | -.137 | .231 | 1.000 | -.696 | .422 |
|  |  | gain | .190 | .230 | 1.000 | -.367 | .747 |
|  | gain | control | -.327 | .228 | .461 | -.879 | .225 |
|  |  | loss | -.190 | .230 | 1.000 | -.747 | .367 |
| Based on estimated marginal means. **p* ≤ .05. | | | | | | | |
| a. Adjustment for multiple comparisons: Bonferroni. | | | | | | | |

Run MATRIX procedure:

**************** PROCESS Procedure for SPSS Version 4.3.1 ****************

Written by Andrew F. Hayes, Ph.D. www.afhayes.com

Documentation available in Hayes (2022). www.guilford.com/p/hayes3

**************************************************************************

Model : CUSTOM

Y : Brst_Int

X : ExpCond

M1 : MsgEmts

M2 : InfoAcpt

M3 : Brst_Att

M4 : Bott_Att

W : Cselfeff

Sample

Size: 167

Coding of categorical X variable for analysis:

ExpCond X1 X2

1.000 .000 .000

2.000 1.000 .000

3.000 .000 1.000

**************************************************************************

OUTCOME VARIABLE:

MsgEmts

Model Summary

R R-sq MSE F df1 df2 p

.777 .604 .482 49.072 5.000 161.000 .000

Model

coeff se t p LLCI ULCI

constant 3.675 .094 39.165 .000 3.489 3.860

X1 -1.368 .133 -10.278 .000 -1.631 -1.105

X2 .289 .133 2.179 .031 .027 .551

Cselfeff -.097 .087 -1.111 .268 -.269 .075

Int_1 .506 .145 3.496 .001 .220 .792

Int_2 .747 .140 5.317 .000 .469 1.024

Product terms key:

Int_1 : X1 x Cselfeff

Int_2 : X2 x Cselfeff

Test(s) of highest order unconditional interaction(s):

R2-chng F df1 df2 p

X*W .076 15.455 2.000 161.000 .000

----------

Focal predict: ExpCond (X)

Mod var: Cselfeff (W)

Conditional effects of the focal predictor at values of the moderator(s):

Moderator value(s):

Cselfeff -.926

Effect se t p LLCI ULCI

X1 -1.837 .183 -10.060 .000 -2.197 -1.476

X2 -.402 .189 -2.125 .035 -.776 -.029

Test of equality of conditional means

F df1 df2 p

52.232 2.000 161.000 .000

Estimated conditional means being compared:

ExpCond MsgEmts

1.000 3.764

2.000 1.928

3.000 3.362

----------

Moderator value(s):

Cselfeff .000

Effect se t p LLCI ULCI

X1 -1.368 .133 -10.278 .000 -1.631 -1.105

X2 .289 .133 2.179 .031 .027 .551

Test of equality of conditional means

F df1 df2 p

88.344 2.000 161.000 .000

Estimated conditional means being compared:

ExpCond MsgEmts

1.000 3.675

2.000 2.306

3.000 3.964

----------

Moderator value(s):

Cselfeff .926

Effect se t p LLCI ULCI

X1 -.900 .195 -4.616 .000 -1.285 -.515

X2 .980 .182 5.384 .000 .620 1.339

Test of equality of conditional means

F df1 df2 p

49.730 2.000 161.000 .000

Estimated conditional means being compared:

ExpCond MsgEmts

1.000 3.585

2.000 2.685

3.000 4.565

Data for visualizing the conditional effect of the focal predictor:

Paste text below into a SPSS syntax window and execute to produce plot.

DATA LIST FREE/

ExpCond Cselfeff MsgEmts .

BEGIN DATA.

1.000 -.926 3.764

2.000 -.926 1.928

3.000 -.926 3.362

1.000 .000 3.675

2.000 .000 2.306

3.000 .000 3.964

1.000 .926 3.585

2.000 .926 2.685

3.000 .926 4.565

END DATA.

GRAPH/SCATTERPLOT=

Cselfeff WITH MsgEmts BY ExpCond .

**************************************************************************

OUTCOME VARIABLE:

InfoAcpt

Model Summary

R R-sq MSE F df1 df2 p

.782 .612 .400 42.040 6.000 160.000 .000

Model

coeff se t p LLCI ULCI

constant 1.548 .277 5.581 .000 1.000 2.096

X1 .153 .156 .978 .330 -.156 .461

X2 .883 .123 7.205 .000 .641 1.125

MsgEmts .395 .072 5.500 .000 .253 .537

Cselfeff .038 .080 .480 .632 -.119 .196

Int_1 .256 .137 1.870 .063 -.014 .526

Int_2 .318 .139 2.290 .023 .044 .592

Product terms key:

Int_1 : X1 x Cselfeff

Int_2 : X2 x Cselfeff

Test(s) of highest order unconditional interaction(s):

R2-chng F df1 df2 p

X*W .015 3.092 2.000 160.000 .048

----------

Focal predict: ExpCond (X)

Mod var: Cselfeff (W)

Conditional effects of the focal predictor at values of the moderator(s):

Moderator value(s):

Cselfeff -.926

Effect se t p LLCI ULCI

X1 -.084 .212 -.396 .692 -.503 .335

X2 .589 .175 3.370 .001 .244 .934

Test of equality of conditional means

F df1 df2 p

7.439 2.000 160.000 .001

Estimated conditional means being compared:

ExpCond InfoAcpt

1.000 2.845

2.000 2.761

3.000 3.434

----------

Moderator value(s):

Cselfeff .000

Effect se t p LLCI ULCI

X1 .153 .156 .978 .330 -.156 .461

X2 .883 .123 7.205 .000 .641 1.125

Test of equality of conditional means

F df1 df2 p

26.508 2.000 160.000 .000

Estimated conditional means being compared:

ExpCond InfoAcpt

1.000 2.880

2.000 3.033

3.000 3.764

----------

Moderator value(s):

Cselfeff .926

Effect se t p LLCI ULCI

X1 .389 .189 2.059 .041 .016 .763

X2 1.177 .180 6.534 .000 .821 1.533

Test of equality of conditional means

F df1 df2 p

21.356 2.000 160.000 .000

Estimated conditional means being compared:

ExpCond InfoAcpt

1.000 2.916

2.000 3.305

3.000 4.093

Data for visualizing the conditional effect of the focal predictor:

Paste text below into a SPSS syntax window and execute to produce plot.

DATA LIST FREE/

ExpCond Cselfeff InfoAcpt .

BEGIN DATA.

1.000 -.926 2.845

2.000 -.926 2.761

3.000 -.926 3.434

1.000 .000 2.880

2.000 .000 3.033

3.000 .000 3.764

1.000 .926 2.916

2.000 .926 3.305

3.000 .926 4.093

END DATA.

GRAPH/SCATTERPLOT=

Cselfeff WITH InfoAcpt BY ExpCond .

**************************************************************************

OUTCOME VARIABLE:

Brst_Att

Model Summary

R R-sq MSE F df1 df2 p

.681 .463 .355 19.615 7.000 159.000 .000

Model

coeff se t p LLCI ULCI

constant 2.941 .286 10.291 .000 2.377 3.506

X1 .182 .148 1.234 .219 -.109 .474

X2 -.056 .133 -.423 .673 -.319 .206

MsgEmts .052 .074 .700 .485 -.094 .197

InfoAcpt .230 .075 3.092 .002 .083 .378

Cselfeff .542 .075 7.206 .000 .394 .691

Int_1 -.116 .130 -.893 .373 -.374 .141

Int_2 -.199 .133 -1.495 .137 -.461 .064

Product terms key:

Int_1 : X1 x Cselfeff

Int_2 : X2 x Cselfeff

Test(s) of highest order unconditional interaction(s):

R2-chng F df1 df2 p

X*W .008 1.148 2.000 159.000 .320

----------

Focal predict: ExpCond (X)

Mod var: Cselfeff (W)

Data for visualizing the conditional effect of the focal predictor:

Paste text below into a SPSS syntax window and execute to produce plot.

DATA LIST FREE/

ExpCond Cselfeff Brst_Att .

BEGIN DATA.

1.000 -.926 3.362

2.000 -.926 3.652

3.000 -.926 3.490

1.000 .000 3.864

2.000 .000 4.046

3.000 .000 3.808

1.000 .926 4.366

2.000 .926 4.440

3.000 .926 4.126

END DATA.

GRAPH/SCATTERPLOT=

Cselfeff WITH Brst_Att BY ExpCond .

**************************************************************************

OUTCOME VARIABLE:

Bott_Att

Model Summary

R R-sq MSE F df1 df2 p

.580 .336 .609 11.496 7.000 159.000 .000

Model

coeff se t p LLCI ULCI

constant 4.337 .374 11.596 .000 3.599 5.076

X1 .055 .193 .287 .775 -.326 .437

X2 .507 .174 2.915 .004 .164 .851

MsgEmts -.074 .097 -.765 .446 -.265 .117

InfoAcpt -.292 .098 -2.997 .003 -.485 -.100

Cselfeff -.513 .098 -5.209 .000 -.707 -.318

Int_1 .077 .171 .452 .652 -.260 .414

Int_2 .228 .174 1.312 .191 -.115 .572

Product terms key:

Int_1 : X1 x Cselfeff

Int_2 : X2 x Cselfeff

Test(s) of highest order unconditional interaction(s):

R2-chng F df1 df2 p

X*W .007 .873 2.000 159.000 .420

----------

Focal predict: ExpCond (X)

Mod var: Cselfeff (W)

Data for visualizing the conditional effect of the focal predictor:

Paste text below into a SPSS syntax window and execute to produce plot.

DATA LIST FREE/

ExpCond Cselfeff Bott_Att .

BEGIN DATA.

1.000 -.926 3.613

2.000 -.926 3.597

3.000 -.926 3.909

1.000 .000 3.139

2.000 .000 3.194

3.000 .000 3.646

1.000 .926 2.664

2.000 .926 2.791

3.000 .926 3.382

END DATA.

GRAPH/SCATTERPLOT=

Cselfeff WITH Bott_Att BY ExpCond .

**************************************************************************

OUTCOME VARIABLE:

Brst_Int

Model Summary

R R-sq MSE F df1 df2 p

.877 .768 .421 57.840 9.000 157.000 .000

Model

coeff se t p LLCI ULCI

constant .712 .511 1.392 .166 -.298 1.722

X1 -.505 .161 -3.128 .002 -.824 -.186

X2 -.255 .149 -1.718 .088 -.549 .038

MsgEmts -.224 .081 -2.783 .006 -.383 -.065

InfoAcpt .126 .085 1.482 .140 -.042 .295

Brst_Att 1.000 .087 11.513 .000 .828 1.172

Bott_Att -.135 .066 -2.035 .044 -.266 -.004

Cselfeff .350 .099 3.549 .001 .155 .545

Int_1 .064 .142 .452 .652 -.217 .345

Int_2 .206 .146 1.409 .161 -.083 .495

Product terms key:

Int_1 : X1 x Cselfeff

Int_2 : X2 x Cselfeff

Test(s) of highest order unconditional interaction(s):

R2-chng F df1 df2 p

X*W .003 1.015 2.000 157.000 .365

----------

Focal predict: ExpCond (X)

Mod var: Cselfeff (W)

Data for visualizing the conditional effect of the focal predictor:

Paste text below into a SPSS syntax window and execute to produce plot.

DATA LIST FREE/

ExpCond Cselfeff Brst_Int .

BEGIN DATA.

1.000 -.926 3.484

2.000 -.926 2.919

3.000 -.926 3.038

1.000 .000 3.808

2.000 .000 3.303

3.000 .000 3.553

1.000 .926 4.132

2.000 .926 3.686

3.000 .926 4.067

END DATA.

GRAPH/SCATTERPLOT=

Cselfeff WITH Brst_Int BY ExpCond .

****************** DIRECT AND INDIRECT EFFECTS OF X ON Y *****************

Relative conditional direct effects of X on Y

Cselfeff Effect se t p LLCI ULCI

X1 -.926 -.565 .219 -2.574 .011 -.998 -.131

X1 .000 -.505 .161 -3.128 .002 -.824 -.186

X1 .926 -.446 .197 -2.264 .025 -.834 -.057

X2 -.926 -.446 .187 -2.384 .018 -.815 -.076

X2 .000 -.255 .149 -1.718 .088 -.549 .038

X2 .926 -.065 .214 -.302 .763 -.487 .358

Relative conditional indirect effects of X on Y:

INDIRECT EFFECT:

ExpCond -> MsgEmts -> Brst_Int

Cselfeff Effect BootSE BootLLCI BootULCI

X1 -.926 .412 .146 .114 .694

X1 .000 .307 .112 .084 .523

X1 .926 .202 .091 .047 .399

Index of moderated mediation:

Index BootSE BootLLCI BootULCI

Cselfeff -.113 .051 -.224 -.023

Cselfeff Effect BootSE BootLLCI BootULCI

X2 -.926 .090 .066 -.007 .245

X2 .000 -.065 .036 -.143 -.003

X2 .926 -.220 .084 -.391 -.061

Index of moderated mediation:

Index BootSE BootLLCI BootULCI

Cselfeff -.167 .071 -.326 -.043

INDIRECT EFFECT:

ExpCond -> InfoAcpt -> Brst_Int

Cselfeff Effect BootSE BootLLCI BootULCI

X1 -.926 -.011 .043 -.131 .053

X1 .000 .019 .029 -.035 .084

X1 .926 .049 .047 -.019 .165

Index of moderated mediation:

Index BootSE BootLLCI BootULCI

Cselfeff .032 .038 -.012 .133

Cselfeff Effect BootSE BootLLCI BootULCI

X2 -.926 .074 .059 -.024 .213

X2 .000 .112 .084 -.037 .293

X2 .926 .149 .113 -.048 .392

Index of moderated mediation:

Index BootSE BootLLCI BootULCI

Cselfeff .040 .035 -.013 .127

INDIRECT EFFECT:

ExpCond -> Brst_Att -> Brst_Int

Cselfeff Effect BootSE BootLLCI BootULCI

X1 -.926 .290 .211 -.113 .707

X1 .000 .182 .150 -.102 .495

X1 .926 .074 .161 -.214 .421

Index of moderated mediation:

Index BootSE BootLLCI BootULCI

Cselfeff -.116 .122 -.343 .133

Cselfeff Effect BootSE BootLLCI BootULCI

X2 -.926 .128 .186 -.242 .497

X2 .000 -.056 .139 -.338 .216

X2 .926 -.240 .166 -.582 .075

Index of moderated mediation:

Index BootSE BootLLCI BootULCI

Cselfeff -.199 .116 -.445 .015

INDIRECT EFFECT:

ExpCond -> Bott_Att -> Brst_Int

Cselfeff Effect BootSE BootLLCI BootULCI

X1 -.926 .002 .035 -.075 .073

X1 .000 -.007 .027 -.069 .044

X1 .926 -.017 .042 -.115 .061

Index of moderated mediation:

Index BootSE BootLLCI BootULCI

Cselfeff -.010 .030 -.076 .051

Cselfeff Effect BootSE BootLLCI BootULCI

X2 -.926 -.040 .036 -.127 .010

X2 .000 -.069 .042 -.161 .002

X2 .926 -.097 .061 -.231 .003

Index of moderated mediation:

Index BootSE BootLLCI BootULCI

Cselfeff -.031 .029 -.095 .017

INDIRECT EFFECT:

ExpCond -> MsgEmts -> InfoAcpt -> Brst_Int

Cselfeff Effect BootSE BootLLCI BootULCI

X1 -.926 -.092 .065 -.223 .034

X1 .000 -.068 .048 -.165 .026

X1 .926 -.045 .034 -.118 .016

Index of moderated mediation:

Index BootSE BootLLCI BootULCI

Cselfeff .025 .020 -.009 .072

Cselfeff Effect BootSE BootLLCI BootULCI

X2 -.926 -.020 .020 -.068 .009

X2 .000 .014 .013 -.005 .044

X2 .926 .049 .036 -.018 .125

Index of moderated mediation:

Index BootSE BootLLCI BootULCI

Cselfeff .037 .029 -.014 .099

INDIRECT EFFECT:

ExpCond -> MsgEmts -> Brst_Att -> Brst_Int

Cselfeff Effect BootSE BootLLCI BootULCI

X1 -.926 -.095 .158 -.414 .206

X1 .000 -.071 .118 -.314 .152

X1 .926 -.047 .082 -.226 .098

Index of moderated mediation:

Index BootSE BootLLCI BootULCI

Cselfeff .026 .046 -.061 .124

Cselfeff Effect BootSE BootLLCI BootULCI

X2 -.926 -.021 .045 -.137 .045

X2 .000 .015 .027 -.040 .070

X2 .926 .051 .086 -.115 .226

Index of moderated mediation:

Index BootSE BootLLCI BootULCI

Cselfeff .039 .068 -.083 .188

INDIRECT EFFECT:

ExpCond -> MsgEmts -> Bott_Att -> Brst_Int

Cselfeff Effect BootSE BootLLCI BootULCI

X1 -.926 -.018 .028 -.084 .032

X1 .000 -.014 .021 -.065 .023

X1 .926 -.009 .015 -.048 .014

Index of moderated mediation:

Index BootSE BootLLCI BootULCI

Cselfeff .005 .008 -.010 .024

Cselfeff Effect BootSE BootLLCI BootULCI

X2 -.926 -.004 .008 -.023 .009

X2 .000 .003 .005 -.005 .015

X2 .926 .010 .015 -.019 .043

Index of moderated mediation:

Index BootSE BootLLCI BootULCI

Cselfeff .007 .012 -.015 .034

INDIRECT EFFECT:

ExpCond -> InfoAcpt -> Brst_Att -> Brst_Int

Cselfeff Effect BootSE BootLLCI BootULCI

X1 -.926 -.019 .065 -.160 .108

X1 .000 .035 .045 -.045 .134

X1 .926 .090 .057 -.002 .218

Index of moderated mediation:

Index BootSE BootLLCI BootULCI

Cselfeff .059 .046 -.014 .168

Cselfeff Effect BootSE BootLLCI BootULCI

X2 -.926 .136 .059 .036 .268

X2 .000 .203 .075 .070 .365

X2 .926 .271 .099 .095 .489

Index of moderated mediation:

Index BootSE BootLLCI BootULCI

Cselfeff .073 .036 .016 .158

INDIRECT EFFECT:

ExpCond -> InfoAcpt -> Bott_Att -> Brst_Int

Cselfeff Effect BootSE BootLLCI BootULCI

X1 -.926 -.003 .013 -.035 .020

X1 .000 .006 .009 -.009 .027

X1 .926 .015 .013 -.003 .047

Index of moderated mediation:

Index BootSE BootLLCI BootULCI

Cselfeff .010 .010 -.003 .036

Cselfeff Effect BootSE BootLLCI BootULCI

X2 -.926 .023 .017 -.001 .063

X2 .000 .035 .023 -.001 .088

X2 .926 .046 .031 -.002 .118

Index of moderated mediation:

Index BootSE BootLLCI BootULCI

Cselfeff .013 .010 -.001 .035

INDIRECT EFFECT:

ExpCond -> MsgEmts -> InfoAcpt -> Brst_Att -> Brst_Int

Cselfeff Effect BootSE BootLLCI BootULCI

X1 -.926 -.167 .067 -.318 -.055

X1 .000 -.125 .049 -.236 -.040

X1 .926 -.082 .038 -.171 -.022

Index of moderated mediation:

Index BootSE BootLLCI BootULCI

Cselfeff .046 .024 .010 .102

Cselfeff Effect BootSE BootLLCI BootULCI

X2 -.926 -.037 .028 -.105 .002

X2 .000 .026 .016 .002 .062

X2 .926 .089 .037 .029 .179

Index of moderated mediation:

Index BootSE BootLLCI BootULCI

Cselfeff .068 .031 .020 .144

INDIRECT EFFECT:

ExpCond -> MsgEmts -> InfoAcpt -> Bott_Att -> Brst_Int

Cselfeff Effect BootSE BootLLCI BootULCI

X1 -.926 -.029 .019 -.072 .001

X1 .000 -.021 .014 -.054 .001

X1 .926 -.014 .010 -.036 .001

Index of moderated mediation:

Index BootSE BootLLCI BootULCI

Cselfeff .008 .006 .000 .023

Cselfeff Effect BootSE BootLLCI BootULCI

X2 -.926 -.006 .006 -.023 .001

X2 .000 .005 .004 .000 .013

X2 .926 .015 .010 .000 .039

Index of moderated mediation:

Index BootSE BootLLCI BootULCI

Cselfeff .012 .008 .000 .032

*********************** ANALYSIS NOTES AND ERRORS ************************

Level of confidence for all confidence intervals in output:

95.0000

Number of bootstrap samples for percentile bootstrap confidence intervals:

5000

W values in conditional tables are the mean and +/- SD from the mean.

------ END MATRIX -----

Run MATRIX procedure:

**************** PROCESS Procedure for SPSS Version 4.3.1 ****************

Written by Andrew F. Hayes, Ph.D. www.afhayes.com

Documentation available in Hayes (2022). www.guilford.com/p/hayes3

**************************************************************************

Model : CUSTOM

Y : Bott_Int

X : ExpCond

M1 : MsgEmts

M2 : InfoAcpt

M3 : Brst_Att

M4 : Bott_Att

W : Cselfeff

Sample

Size: 167

Coding of categorical X variable for analysis:

ExpCond X1 X2

1.000 .000 .000

2.000 1.000 .000

3.000 .000 1.000

**************************************************************************

OUTCOME VARIABLE:

MsgEmts

Model Summary

R R-sq MSE F df1 df2 p

.777 .604 .482 49.072 5.000 161.000 .000

Model

coeff se t p LLCI ULCI

constant 3.675 .094 39.165 .000 3.489 3.860

X1 -1.368 .133 -10.278 .000 -1.631 -1.105

X2 .289 .133 2.179 .031 .027 .551

Cselfeff -.097 .087 -1.111 .268 -.269 .075

Int_1 .506 .145 3.496 .001 .220 .792

Int_2 .747 .140 5.317 .000 .469 1.024

Product terms key:

Int_1 : X1 x Cselfeff

Int_2 : X2 x Cselfeff

Test(s) of highest order unconditional interaction(s):

R2-chng F df1 df2 p

X*W .076 15.455 2.000 161.000 .000

----------

Focal predict: ExpCond (X)

Mod var: Cselfeff (W)

Conditional effects of the focal predictor at values of the moderator(s):

Moderator value(s):

Cselfeff -.926

Effect se t p LLCI ULCI

X1 -1.837 .183 -10.060 .000 -2.197 -1.476

X2 -.402 .189 -2.125 .035 -.776 -.029

Test of equality of conditional means

F df1 df2 p

52.232 2.000 161.000 .000

Estimated conditional means being compared:

ExpCond MsgEmts

1.000 3.764

2.000 1.928

3.000 3.362

----------

Moderator value(s):

Cselfeff .000

Effect se t p LLCI ULCI

X1 -1.368 .133 -10.278 .000 -1.631 -1.105

X2 .289 .133 2.179 .031 .027 .551

Test of equality of conditional means

F df1 df2 p

88.344 2.000 161.000 .000

Estimated conditional means being compared:

ExpCond MsgEmts

1.000 3.675

2.000 2.306

3.000 3.964

----------

Moderator value(s):

Cselfeff .926

Effect se t p LLCI ULCI

X1 -.900 .195 -4.616 .000 -1.285 -.515

X2 .980 .182 5.384 .000 .620 1.339

Test of equality of conditional means

F df1 df2 p

49.730 2.000 161.000 .000

Estimated conditional means being compared:

ExpCond MsgEmts

1.000 3.585

2.000 2.685

3.000 4.565

Data for visualizing the conditional effect of the focal predictor:

Paste text below into a SPSS syntax window and execute to produce plot.

DATA LIST FREE/

ExpCond Cselfeff MsgEmts .

BEGIN DATA.

1.000 -.926 3.764

2.000 -.926 1.928

3.000 -.926 3.362

1.000 .000 3.675

2.000 .000 2.306

3.000 .000 3.964

1.000 .926 3.585

2.000 .926 2.685

3.000 .926 4.565

END DATA.

GRAPH/SCATTERPLOT=

Cselfeff WITH MsgEmts BY ExpCond .

**************************************************************************

OUTCOME VARIABLE:

InfoAcpt

Model Summary

R R-sq MSE F df1 df2 p

.782 .612 .400 42.040 6.000 160.000 .000

Model

coeff se t p LLCI ULCI

constant 1.548 .277 5.581 .000 1.000 2.096

X1 .153 .156 .978 .330 -.156 .461

X2 .883 .123 7.205 .000 .641 1.125

MsgEmts .395 .072 5.500 .000 .253 .537

Cselfeff .038 .080 .480 .632 -.119 .196

Int_1 .256 .137 1.870 .063 -.014 .526

Int_2 .318 .139 2.290 .023 .044 .592

Product terms key:

Int_1 : X1 x Cselfeff

Int_2 : X2 x Cselfeff

Test(s) of highest order unconditional interaction(s):

R2-chng F df1 df2 p

X*W .015 3.092 2.000 160.000 .048

----------

Focal predict: ExpCond (X)

Mod var: Cselfeff (W)

Conditional effects of the focal predictor at values of the moderator(s):

Moderator value(s):

Cselfeff -.926

Effect se t p LLCI ULCI

X1 -.084 .212 -.396 .692 -.503 .335

X2 .589 .175 3.370 .001 .244 .934

Test of equality of conditional means

F df1 df2 p

7.439 2.000 160.000 .001

Estimated conditional means being compared:

ExpCond InfoAcpt

1.000 2.845

2.000 2.761

3.000 3.434

----------

Moderator value(s):

Cselfeff .000

Effect se t p LLCI ULCI

X1 .153 .156 .978 .330 -.156 .461

X2 .883 .123 7.205 .000 .641 1.125

Test of equality of conditional means

F df1 df2 p

26.508 2.000 160.000 .000

Estimated conditional means being compared:

ExpCond InfoAcpt

1.000 2.880

2.000 3.033

3.000 3.764

----------

Moderator value(s):

Cselfeff .926

Effect se t p LLCI ULCI

X1 .389 .189 2.059 .041 .016 .763

X2 1.177 .180 6.534 .000 .821 1.533

Test of equality of conditional means

F df1 df2 p

21.356 2.000 160.000 .000

Estimated conditional means being compared:

ExpCond InfoAcpt

1.000 2.916

2.000 3.305

3.000 4.093

Data for visualizing the conditional effect of the focal predictor:

Paste text below into a SPSS syntax window and execute to produce plot.

DATA LIST FREE/

ExpCond Cselfeff InfoAcpt .

BEGIN DATA.

1.000 -.926 2.845

2.000 -.926 2.761

3.000 -.926 3.434

1.000 .000 2.880

2.000 .000 3.033

3.000 .000 3.764

1.000 .926 2.916

2.000 .926 3.305

3.000 .926 4.093

END DATA.

GRAPH/SCATTERPLOT=

Cselfeff WITH InfoAcpt BY ExpCond .

**************************************************************************

OUTCOME VARIABLE:

Brst_Att

Model Summary

R R-sq MSE F df1 df2 p

.681 .463 .355 19.615 7.000 159.000 .000

Model

coeff se t p LLCI ULCI

constant 2.941 .286 10.291 .000 2.377 3.506

X1 .182 .148 1.234 .219 -.109 .474

X2 -.056 .133 -.423 .673 -.319 .206

MsgEmts .052 .074 .700 .485 -.094 .197

InfoAcpt .230 .075 3.092 .002 .083 .378

Cselfeff .542 .075 7.206 .000 .394 .691

Int_1 -.116 .130 -.893 .373 -.374 .141

Int_2 -.199 .133 -1.495 .137 -.461 .064

Product terms key:

Int_1 : X1 x Cselfeff

Int_2 : X2 x Cselfeff

Test(s) of highest order unconditional interaction(s):

R2-chng F df1 df2 p

X*W .008 1.148 2.000 159.000 .320

----------

Focal predict: ExpCond (X)

Mod var: Cselfeff (W)

Data for visualizing the conditional effect of the focal predictor:

Paste text below into a SPSS syntax window and execute to produce plot.

DATA LIST FREE/

ExpCond Cselfeff Brst_Att .

BEGIN DATA.

1.000 -.926 3.362

2.000 -.926 3.652

3.000 -.926 3.490

1.000 .000 3.864

2.000 .000 4.046

3.000 .000 3.808

1.000 .926 4.366

2.000 .926 4.440

3.000 .926 4.126

END DATA.

GRAPH/SCATTERPLOT=

Cselfeff WITH Brst_Att BY ExpCond .

**************************************************************************

OUTCOME VARIABLE:

Bott_Att

Model Summary

R R-sq MSE F df1 df2 p

.580 .336 .609 11.496 7.000 159.000 .000

Model

coeff se t p LLCI ULCI

constant 4.337 .374 11.596 .000 3.599 5.076

X1 .055 .193 .287 .775 -.326 .437

X2 .507 .174 2.915 .004 .164 .851

MsgEmts -.074 .097 -.765 .446 -.265 .117

InfoAcpt -.292 .098 -2.997 .003 -.485 -.100

Cselfeff -.513 .098 -5.209 .000 -.707 -.318

Int_1 .077 .171 .452 .652 -.260 .414

Int_2 .228 .174 1.312 .191 -.115 .572

Product terms key:

Int_1 : X1 x Cselfeff

Int_2 : X2 x Cselfeff

Test(s) of highest order unconditional interaction(s):

R2-chng F df1 df2 p

X*W .007 .873 2.000 159.000 .420

----------

Focal predict: ExpCond (X)

Mod var: Cselfeff (W)

Data for visualizing the conditional effect of the focal predictor:

Paste text below into a SPSS syntax window and execute to produce plot.

DATA LIST FREE/

ExpCond Cselfeff Bott_Att .

BEGIN DATA.

1.000 -.926 3.613

2.000 -.926 3.597

3.000 -.926 3.909

1.000 .000 3.139

2.000 .000 3.194

3.000 .000 3.646

1.000 .926 2.664

2.000 .926 2.791

3.000 .926 3.382

END DATA.

GRAPH/SCATTERPLOT=

Cselfeff WITH Bott_Att BY ExpCond .

**************************************************************************

OUTCOME VARIABLE:

Bott_Int

Model Summary

R R-sq MSE F df1 df2 p

.872 .760 .374 55.156 9.000 157.000 .000

Model

coeff se t p LLCI ULCI

constant 3.874 .482 8.040 .000 2.923 4.826

X1 .063 .152 .412 .681 -.238 .363

X2 .059 .140 .425 .672 -.217 .336

MsgEmts .118 .076 1.554 .122 -.032 .268

InfoAcpt -.049 .080 -.612 .541 -.208 .109

Brst_Att -.775 .082 -9.471 .000 -.937 -.613

Bott_Att .404 .063 6.459 .000 .280 .527

Cselfeff -.297 .093 -3.195 .002 -.480 -.113

Int_1 .044 .134 .327 .744 -.221 .309

Int_2 -.085 .138 -.618 .538 -.357 .187

Product terms key:

Int_1 : X1 x Cselfeff

Int_2 : X2 x Cselfeff

Test(s) of highest order unconditional interaction(s):

R2-chng F df1 df2 p

X*W .001 .424 2.000 157.000 .655

----------

Focal predict: ExpCond (X)

Mod var: Cselfeff (W)

Data for visualizing the conditional effect of the focal predictor:

Paste text below into a SPSS syntax window and execute to produce plot.

DATA LIST FREE/

ExpCond Cselfeff Bott_Int .

BEGIN DATA.

1.000 -.926 2.720

2.000 -.926 2.742

3.000 -.926 2.858

1.000 .000 2.445

2.000 .000 2.508

3.000 .000 2.505

1.000 .926 2.171

2.000 .926 2.274

3.000 .926 2.151

END DATA.

GRAPH/SCATTERPLOT=

Cselfeff WITH Bott_Int BY ExpCond .

****************** DIRECT AND INDIRECT EFFECTS OF X ON Y *****************

Relative conditional direct effects of X on Y

Cselfeff Effect se t p LLCI ULCI

X1 -.926 .022 .207 .107 .915 -.386 .430

X1 .000 .063 .152 .412 .681 -.238 .363

X1 .926 .103 .185 .557 .578 -.263 .470

X2 -.926 .138 .176 .784 .434 -.210 .486

X2 .000 .059 .140 .425 .672 -.217 .336

X2 .926 -.019 .202 -.096 .924 -.417 .379

Relative conditional indirect effects of X on Y:

INDIRECT EFFECT:

ExpCond -> MsgEmts -> Bott_Int

Cselfeff Effect BootSE BootLLCI BootULCI

X1 -.926 -.217 .141 -.491 .068

X1 .000 -.161 .107 -.373 .049

X1 .926 -.106 .078 -.279 .027

Index of moderated mediation:

Index BootSE BootLLCI BootULCI

Cselfeff .060 .042 -.018 .150

Cselfeff Effect BootSE BootLLCI BootULCI

X2 -.926 -.047 .043 -.154 .019

X2 .000 .034 .028 -.011 .098

X2 .926 .116 .075 -.037 .266

Index of moderated mediation:

Index BootSE BootLLCI BootULCI

Cselfeff .088 .059 -.028 .208

INDIRECT EFFECT:

ExpCond -> InfoAcpt -> Bott_Int

Cselfeff Effect BootSE BootLLCI BootULCI

X1 -.926 .004 .028 -.044 .079

X1 .000 -.008 .021 -.061 .032

X1 .926 -.019 .040 -.119 .050

Index of moderated mediation:

Index BootSE BootLLCI BootULCI

Cselfeff -.013 .030 -.090 .035

Cselfeff Effect BootSE BootLLCI BootULCI

X2 -.926 -.029 .056 -.150 .072

X2 .000 -.043 .081 -.212 .106

X2 .926 -.058 .107 -.276 .143

Index of moderated mediation:

Index BootSE BootLLCI BootULCI

Cselfeff -.016 .030 -.080 .042

INDIRECT EFFECT:

ExpCond -> Brst_Att -> Bott_Int

Cselfeff Effect BootSE BootLLCI BootULCI

X1 -.926 -.225 .166 -.558 .094

X1 .000 -.141 .116 -.374 .082

X1 .926 -.058 .124 -.314 .176

Index of moderated mediation:

Index BootSE BootLLCI BootULCI

Cselfeff .090 .096 -.098 .284

Cselfeff Effect BootSE BootLLCI BootULCI

X2 -.926 -.099 .143 -.372 .182

X2 .000 .044 .110 -.163 .270

X2 .926 .186 .136 -.064 .471

Index of moderated mediation:

Index BootSE BootLLCI BootULCI

Cselfeff .154 .094 -.022 .353

INDIRECT EFFECT:

ExpCond -> Bott_Att -> Bott_Int

Cselfeff Effect BootSE BootLLCI BootULCI

X1 -.926 -.006 .094 -.197 .173

X1 .000 .022 .067 -.114 .150

X1 .926 .051 .102 -.141 .259

Index of moderated mediation:

Index BootSE BootLLCI BootULCI

Cselfeff .031 .078 -.113 .193

Cselfeff Effect BootSE BootLLCI BootULCI

X2 -.926 .120 .080 -.019 .292

X2 .000 .205 .075 .075 .369

X2 .926 .290 .111 .085 .527

Index of moderated mediation:

Index BootSE BootLLCI BootULCI

Cselfeff .092 .067 -.044 .220

INDIRECT EFFECT:

ExpCond -> MsgEmts -> InfoAcpt -> Bott_Int

Cselfeff Effect BootSE BootLLCI BootULCI

X1 -.926 .036 .065 -.093 .168

X1 .000 .027 .048 -.069 .126

X1 .926 .017 .033 -.045 .090

Index of moderated mediation:

Index BootSE BootLLCI BootULCI

Cselfeff -.010 .019 -.050 .027

Cselfeff Effect BootSE BootLLCI BootULCI

X2 -.926 .008 .018 -.024 .050

X2 .000 -.006 .011 -.030 .016

X2 .926 -.019 .035 -.091 .050

Index of moderated mediation:

Index BootSE BootLLCI BootULCI

Cselfeff -.015 .028 -.072 .039

INDIRECT EFFECT:

ExpCond -> MsgEmts -> Brst_Att -> Bott_Int

Cselfeff Effect BootSE BootLLCI BootULCI

X1 -.926 .074 .124 -.160 .329

X1 .000 .055 .093 -.116 .248

X1 .926 .036 .064 -.077 .177

Index of moderated mediation:

Index BootSE BootLLCI BootULCI

Cselfeff -.020 .036 -.100 .048

Cselfeff Effect BootSE BootLLCI BootULCI

X2 -.926 .016 .035 -.036 .107

X2 .000 -.012 .021 -.057 .031

X2 .926 -.039 .066 -.174 .087

Index of moderated mediation:

Index BootSE BootLLCI BootULCI

Cselfeff -.030 .053 -.145 .066

INDIRECT EFFECT:

ExpCond -> MsgEmts -> Bott_Att -> Bott_Int

Cselfeff Effect BootSE BootLLCI BootULCI

X1 -.926 .055 .073 -.085 .209

X1 .000 .041 .055 -.061 .159

X1 .926 .027 .038 -.037 .118

Index of moderated mediation:

Index BootSE BootLLCI BootULCI

Cselfeff -.015 .021 -.060 .027

Cselfeff Effect BootSE BootLLCI BootULCI

X2 -.926 .012 .019 -.024 .056

X2 .000 -.009 .013 -.041 .014

X2 .926 -.029 .039 -.107 .046

Index of moderated mediation:

Index BootSE BootLLCI BootULCI

Cselfeff -.022 .030 -.082 .038

INDIRECT EFFECT:

ExpCond -> InfoAcpt -> Brst_Att -> Bott_Int

Cselfeff Effect BootSE BootLLCI BootULCI

X1 -.926 .015 .050 -.085 .121

X1 .000 -.027 .035 -.107 .035

X1 .926 -.070 .046 -.174 .000

Index of moderated mediation:

Index BootSE BootLLCI BootULCI

Cselfeff -.046 .035 -.134 .008

Cselfeff Effect BootSE BootLLCI BootULCI

X2 -.926 -.105 .047 -.213 -.029

X2 .000 -.158 .060 -.290 -.053

X2 .926 -.210 .079 -.387 -.072

Index of moderated mediation:

Index BootSE BootLLCI BootULCI

Cselfeff -.057 .028 -.120 -.012

INDIRECT EFFECT:

ExpCond -> InfoAcpt -> Bott_Att -> Bott_Int

Cselfeff Effect BootSE BootLLCI BootULCI

X1 -.926 .010 .035 -.049 .094

X1 .000 -.018 .022 -.062 .029

X1 .926 -.046 .030 -.112 .001

Index of moderated mediation:

Index BootSE BootLLCI BootULCI

Cselfeff -.030 .025 -.092 .005

Cselfeff Effect BootSE BootLLCI BootULCI

X2 -.926 -.070 .034 -.146 -.016

X2 .000 -.104 .044 -.203 -.028

X2 .926 -.139 .059 -.273 -.038

Index of moderated mediation:

Index BootSE BootLLCI BootULCI

Cselfeff -.038 .020 -.085 -.006

INDIRECT EFFECT:

ExpCond -> MsgEmts -> InfoAcpt -> Brst_Att -> Bott_Int

Cselfeff Effect BootSE BootLLCI BootULCI

X1 -.926 .130 .052 .041 .245

X1 .000 .097 .039 .031 .183

X1 .926 .063 .029 .017 .130

Index of moderated mediation:

Index BootSE BootLLCI BootULCI

Cselfeff -.036 .019 -.079 -.007

Cselfeff Effect BootSE BootLLCI BootULCI

X2 -.926 .028 .021 -.001 .081

X2 .000 -.020 .012 -.048 -.001

X2 .926 -.069 .029 -.134 -.021

Index of moderated mediation:

Index BootSE BootLLCI BootULCI

Cselfeff -.053 .024 -.108 -.015

INDIRECT EFFECT:

ExpCond -> MsgEmts -> InfoAcpt -> Bott_Att -> Bott_Int

Cselfeff Effect BootSE BootLLCI BootULCI

X1 -.926 .086 .036 .024 .166

X1 .000 .064 .026 .018 .119

X1 .926 .042 .019 .010 .084

Index of moderated mediation:

Index BootSE BootLLCI BootULCI

Cselfeff -.024 .013 -.056 -.004

Cselfeff Effect BootSE BootLLCI BootULCI

X2 -.926 .019 .014 -.001 .054

X2 .000 -.013 .008 -.032 .000

X2 .926 -.046 .020 -.091 -.012

Index of moderated mediation:

Index BootSE BootLLCI BootULCI

Cselfeff -.035 .017 -.073 -.009

*********************** ANALYSIS NOTES AND ERRORS ************************

Level of confidence for all confidence intervals in output:

95.0000

Number of bootstrap samples for percentile bootstrap confidence intervals:

5000

W values in conditional tables are the mean and +/- SD from the mean.

------ END MATRIX -----

## Main analyses rerun on the US subsample (*N* = 79)

| **Test of Between-Participants Effect** | | | | | | | |
| --- | --- | --- | --- | --- | --- | --- | --- |
| Source | DV | Type III Sum of Squares | *df* | Mean square | *F* | *p* | Partial eta squared |
| Corrected Model | Self-efficacy and PBC | .563^a^ | 2 | .282 | .280 | .756 | .007 |
|  | Emotions associated with messages | 65.147^b^ | 2 | 32.573 | 66.389 | .000 | .636 |
|  | Information acceptance | 35.392^c^ | 2 | 17.696 | 25.396 | .000 | .401 |
|  | Brst_Att | 2.278^d^ | 2 | 1.139 | 1.961 | .148 | .049 |
|  | Bott_Att | .486^e^ | 2 | .243 | .357 | .701 | .009 |
|  | Intention to breastfeed | .451^f^ | 2 | .225 | .155 | .857 | .004 |
|  | Intention to feed formula | 1.083^g^ | 2 | .541 | .393 | .677 | .010 |
| Intercept | Self-efficacy and PBC | 792.466 | 1 | 792.466 | 789.404 | .000 | .912 |
|  | Emotions associated with messages | 957.099 | 1 | 957.099 | 1950.707 | .000 | .963 |
|  | Information acceptance | 905.257 | 1 | 905.257 | 1299.140 | .000 | .945 |
|  | Brst_Att | 1189.277 | 1 | 1189.277 | 2047.267 | .000 | .964 |
|  | Bott_Att | 808.422 | 1 | 808.422 | 1186.092 | .000 | .940 |
|  | Intention to breastfeed | 1189.507 | 1 | 1189.507 | 815.488 | .000 | .915 |
|  | Intention to feed formula | 461.356 | 1 | 461.356 | 334.574 | .000 | .815 |
| ExpCond | Self-efficacy and PBC | .563 | 2 | .282 | .280 | .756 | .007 |
|  | Emotions associated with messages | 65.147 | 2 | 32.573 | 66.389 | .000 | .636 |
|  | Information acceptance | 35.392 | 2 | 17.696 | 25.396 | .000 | .401 |
|  | Brst_Att | 2.278 | 2 | 1.139 | 1.961 | .148 | .049 |
|  | Bott_Att | .486 | 2 | .243 | .357 | .701 | .009 |
|  | Intention to breastfeed | .451 | 2 | .225 | .155 | .857 | .004 |
|  | Intention to feed formula | 1.083 | 2 | .541 | .393 | .677 | .010 |
| Error | Self-efficacy and PBC | 76.295 | 76 | 1.004 |  |  |  |
|  | Emotions associated with messages | 37.289 | 76 | .491 |  |  |  |
|  | Information acceptance | 52.958 | 76 | .697 |  |  |  |
|  | Brst_Att | 44.149 | 76 | .581 |  |  |  |
|  | Bott_Att | 51.800 | 76 | .682 |  |  |  |
|  | Intention to breastfeed | 110.857 | 76 | 1.459 |  |  |  |
|  | Intention to feed formula | 104.799 | 76 | 1.379 |  |  |  |
| Total | Self-efficacy and PBC | 872.222 | 79 |  |  |  |  |
|  | Emotions associated with messages | 1076.028 | 79 |  |  |  |  |
|  | Information acceptance | 994.125 | 79 |  |  |  |  |
|  | Brst_Att | 1240.758 | 79 |  |  |  |  |
|  | Bott_Att | 863.328 | 79 |  |  |  |  |
|  | Intention to breastfeed | 1304.333 | 79 |  |  |  |  |
|  | Intention to feed formula | 567.667 | 79 |  |  |  |  |
| Corrected Total | Self-efficacy and PBC | 76.858 | 78 |  |  |  |  |
|  | Emotions associated with messages | 102.436 | 78 |  |  |  |  |
|  | Information acceptance | 88.350 | 78 |  |  |  |  |
|  | Brst_Att | 46.427 | 78 |  |  |  |  |
|  | Bott_Att | 52.287 | 78 |  |  |  |  |
|  | Intention to breastfeed | 111.308 | 78 |  |  |  |  |
|  | Intention to feed formula | 105.882 | 78 |  |  |  |  |
| a. *R*^2^ = .007 (Adjusted *R*^2^ = -.019) | | | | | | | |
| b. *R*^2^ = .636 (Adjusted *R*^2^ = .626) | | | | | | | |
| c. *R*^2^ = .401 (Adjusted *R*^2^ = .385) | | | | | | | |
| d. *R*^2^ = .049 (Adjusted *R*^2^ = .024) | | | | | | | |
| e. *R*^2^ = .009 (Adjusted *R*^2^ = -.017) | | | | | | | |
| f. *R*^2^ = .004 (Adjusted *R*^2^ = -.022) | | | | | | | |
| g. *R*^2^ = .010 (Adjusted *R*^2^ = -.016) | | | | | | | |

| **Descriptives** | | | | |
| --- | --- | --- | --- | --- |
|  | Experimental condition | *M* | *SD* | *N* |
| Self-efficacy and PBC | control | 3.2024 | 1.05542 | 28 |
|  | loss | 3.0533 | .97980 | 25 |
|  | gain | 3.2564 | .96290 | 26 |
|  | Totale | 3.1730 | .99265 | 79 |
| Emotions associated with messages | control | 3.8750 | .54645 | 28 |
|  | loss | 2.2100 | .67577 | 25 |
|  | gain | 4.3686 | .85478 | 26 |
|  | Totale | 3.5105 | 1.14598 | 79 |
| Information acceptance | control | 3.0000 | .00000 | 28 |
|  | loss | 2.8300 | 1.25773 | 25 |
|  | gain | 4.3365 | .77441 | 26 |
|  | Totale | 3.3861 | 1.06428 | 79 |
| Brst_Att | control | 3.9256 | .70409 | 28 |
|  | loss | 3.6550 | .82544 | 25 |
|  | gain | 4.0721 | .75925 | 26 |
|  | Totale | 3.8882 | .77151 | 79 |
| Bott_Att | control | 3.2813 | .77923 | 28 |
|  | loss | 3.2300 | .75836 | 25 |
|  | gain | 3.0962 | .92959 | 26 |
|  | Totale | 3.2041 | .81875 | 79 |
| Intention to breastfeed | control | 3.8929 | 1.22072 | 28 |
|  | loss | 3.7867 | 1.30838 | 25 |
|  | gain | 3.9744 | 1.08699 | 26 |
|  | Totale | 3.8861 | 1.19458 | 79 |
| Intention to feed formula | control | 2.4286 | 1.06519 | 28 |
|  | loss | 2.5600 | 1.35987 | 25 |
|  | gain | 2.2692 | 1.09146 | 26 |
|  | Totale | 2.4177 | 1.16510 | 79 |

| **Pairwise Comparisons** | | | | | | | |
| --- | --- | --- | --- | --- | --- | --- | --- |
| DV | (I) Experimental condition | (J) Experimental condition | Mean Difference (I-J) | *SE* | *p* ^a^ | 95% CI ^a^ | |
|  |  |  |  |  |  | LL | UL |
| Self-efficacy and PBC | control | loss | .149 | .276 | 1.000 | -.526 | .824 |
|  |  | gain | -.054 | .273 | 1.000 | -.722 | .614 |
|  | loss | control | -.149 | .276 | 1.000 | -.824 | .526 |
|  |  | gain | -.203 | .281 | 1.000 | -.890 | .484 |
|  | gain | control | .054 | .273 | 1.000 | -.614 | .722 |
|  |  | loss | .203 | .281 | 1.000 | -.484 | .890 |
| Emotions associated with messages | control | loss | 1.665^*^ | .193 | .000 | 1.193 | 2.137 |
|  |  | gain | -.494^*^ | .191 | .035 | -.961 | -.027 |
|  | loss | control | -1.665^*^ | .193 | .000 | -2.137 | -1.193 |
|  |  | gain | -2.159^*^ | .196 | .000 | -2.639 | -1.678 |
|  | gain | control | .494^*^ | .191 | .035 | .027 | .961 |
|  |  | loss | 2.159^*^ | .196 | .000 | 1.678 | 2.639 |
| Information acceptance | control | loss | .170 | .230 | 1.000 | -.392 | .732 |
|  |  | gain | -1.337^*^ | .227 | .000 | -1.893 | -.780 |
|  | loss | control | -.170 | .230 | 1.000 | -.732 | .392 |
|  |  | gain | -1.507^*^ | .234 | .000 | -2.079 | -.934 |
|  | gain | control | 1.337^*^ | .227 | .000 | .780 | 1.893 |
|  |  | loss | 1.507^*^ | .234 | .000 | .934 | 2.079 |
| Brst_Att | control | loss | .271 | .210 | .602 | -.243 | .784 |
|  |  | gain | -.146 | .208 | 1.000 | -.655 | .362 |
|  | loss | control | -.271 | .210 | .602 | -.784 | .243 |
|  |  | gain | -.417 | .213 | .163 | -.940 | .106 |
|  | gain | control | .146 | .208 | 1.000 | -.362 | .655 |
|  |  | loss | .417 | .213 | .163 | -.106 | .940 |
| Bott_Att | control | loss | .051 | .227 | 1.000 | -.505 | .607 |
|  |  | gain | .185 | .225 | 1.000 | -.365 | .736 |
|  | loss | control | -.051 | .227 | 1.000 | -.607 | .505 |
|  |  | gain | .134 | .231 | 1.000 | -.432 | .700 |
|  | gain | control | -.185 | .225 | 1.000 | -.736 | .365 |
|  |  | loss | -.134 | .231 | 1.000 | -.700 | .432 |
| Intention to breastfeed | control | loss | .106 | .332 | 1.000 | -.707 | .920 |
|  |  | gain | -.082 | .329 | 1.000 | -.887 | .724 |
|  | loss | control | -.106 | .332 | 1.000 | -.920 | .707 |
|  |  | gain | -.188 | .338 | 1.000 | -1.016 | .641 |
|  | gain | control | .082 | .329 | 1.000 | -.724 | .887 |
|  |  | loss | .188 | .338 | 1.000 | -.641 | 1.016 |
| Intention to feed formula | control | loss | -.131 | .323 | 1.000 | -.922 | .660 |
|  |  | gain | .159 | .320 | 1.000 | -.624 | .942 |
|  | loss | control | .131 | .323 | 1.000 | -.660 | .922 |
|  |  | gain | .291 | .329 | 1.000 | -.514 | 1.096 |
|  | gain | control | -.159 | .320 | 1.000 | -.942 | .624 |
|  |  | loss | -.291 | .329 | 1.000 | -1.096 | .514 |
| Based on estimated marginal means. **p* ≤ .05. | | | | | | | |
| a. Adjustment for multiple comparisons: Bonferroni. | | | | | | | |

Run MATRIX procedure:

**************** PROCESS Procedure for SPSS Version 4.3.1 ****************

Written by Andrew F. Hayes, Ph.D. www.afhayes.com

Documentation available in Hayes (2022). www.guilford.com/p/hayes3

**************************************************************************

Model : CUSTOM

Y : Brst_Int

X : ExpCond

M1 : MsgEmts

M2 : InfoAcpt

M3 : Brst_Att

M4 : Bott_Att

W : Cselfeff

Sample

Size: 79

Coding of categorical X variable for analysis:

ExpCond X1 X2

1.000 .000 .000

2.000 1.000 .000

3.000 .000 1.000

**************************************************************************

OUTCOME VARIABLE:

MsgEmts

Model Summary

R R-sq MSE F df1 df2 p

.829 .687 .439 32.020 5.000 73.000 .000

Model

coeff se t p LLCI ULCI

constant 3.872 .125 30.897 .000 3.622 4.122

X1 -1.650 .183 -9.009 .000 -2.015 -1.285

X2 .459 .181 2.535 .013 .098 .819

Cselfeff .093 .121 .768 .445 -.148 .334

Int_1 .007 .184 .039 .969 -.359 .373

Int_2 .358 .183 1.956 .054 -.007 .724

Product terms key:

Int_1 : X1 x Cselfeff

Int_2 : X2 x Cselfeff

Test(s) of highest order unconditional interaction(s):

R2-chng F df1 df2 p

X*W .020 2.319 2.000 73.000 .106

----------

Focal predict: ExpCond (X)

Mod var: Cselfeff (W)

Data for visualizing the conditional effect of the focal predictor:

Paste text below into a SPSS syntax window and execute to produce plot.

DATA LIST FREE/

ExpCond Cselfeff MsgEmts .

BEGIN DATA.

1.000 -.993 3.780

2.000 -.993 2.123

3.000 -.993 3.883

1.000 .000 3.872

2.000 .000 2.222

3.000 .000 4.331

1.000 .993 3.964

2.000 .993 2.321

3.000 .993 4.779

END DATA.

GRAPH/SCATTERPLOT=

Cselfeff WITH MsgEmts BY ExpCond .

**************************************************************************

OUTCOME VARIABLE:

InfoAcpt

Model Summary

R R-sq MSE F df1 df2 p

.708 .501 .613 12.035 6.000 72.000 .000

Model

coeff se t p LLCI ULCI

constant 1.543 .555 2.779 .007 .436 2.650

X1 .467 .314 1.485 .142 -.160 1.093

X2 1.130 .223 5.069 .000 .685 1.574

MsgEmts .376 .138 2.723 .008 .101 .652

Cselfeff -.035 .143 -.244 .808 -.321 .251

Int_1 .130 .217 .600 .550 -.302 .562

Int_2 .275 .222 1.240 .219 -.167 .718

Product terms key:

Int_1 : X1 x Cselfeff

Int_2 : X2 x Cselfeff

Test(s) of highest order unconditional interaction(s):

R2-chng F df1 df2 p

X*W .011 .772 2.000 72.000 .466

----------

Focal predict: ExpCond (X)

Mod var: Cselfeff (W)

Data for visualizing the conditional effect of the focal predictor:

Paste text below into a SPSS syntax window and execute to produce plot.

DATA LIST FREE/

ExpCond Cselfeff InfoAcpt .

BEGIN DATA.

1.000 -.993 2.899

2.000 -.993 3.236

3.000 -.993 3.755

1.000 .000 2.864

2.000 .000 3.331

3.000 .000 3.994

1.000 .993 2.829

2.000 .993 3.425

3.000 .993 4.232

END DATA.

GRAPH/SCATTERPLOT=

Cselfeff WITH InfoAcpt BY ExpCond .

**************************************************************************

OUTCOME VARIABLE:

Brst_Att

Model Summary

R R-sq MSE F df1 df2 p

.754 .568 .282 13.348 7.000 71.000 .000

Model

coeff se t p LLCI ULCI

constant 2.457 .397 6.195 .000 1.666 3.248

X1 .017 .217 .077 .939 -.415 .449

X2 -.391 .176 -2.217 .030 -.742 -.039

MsgEmts .101 .099 1.024 .309 -.096 .297

InfoAcpt .355 .080 4.435 .000 .195 .514

Cselfeff .450 .097 4.629 .000 .256 .645

Int_1 -.068 .147 -.462 .645 -.362 .226

Int_2 -.134 .152 -.879 .383 -.437 .170

Product terms key:

Int_1 : X1 x Cselfeff

Int_2 : X2 x Cselfeff

Test(s) of highest order unconditional interaction(s):

R2-chng F df1 df2 p

X*W .005 .390 2.000 71.000 .678

----------

Focal predict: ExpCond (X)

Mod var: Cselfeff (W)

Data for visualizing the conditional effect of the focal predictor:

Paste text below into a SPSS syntax window and execute to produce plot.

DATA LIST FREE/

ExpCond Cselfeff Brst_Att .

BEGIN DATA.

1.000 -.993 3.565

2.000 -.993 3.650

3.000 -.993 3.308

1.000 .000 4.013

2.000 .000 4.029

3.000 .000 3.622

1.000 .993 4.460

2.000 .993 4.409

3.000 .993 3.936

END DATA.

GRAPH/SCATTERPLOT=

Cselfeff WITH Brst_Att BY ExpCond .

**************************************************************************

OUTCOME VARIABLE:

Bott_Att

Model Summary

R R-sq MSE F df1 df2 p

.575 .330 .493 5.004 7.000 71.000 .000

Model

coeff se t p LLCI ULCI

constant 4.479 .524 8.545 .000 3.434 5.524

X1 -.361 .286 -1.262 .211 -.932 .209

X2 .199 .233 .853 .397 -.266 .663

MsgEmts -.128 .130 -.984 .329 -.388 .132

InfoAcpt -.230 .106 -2.177 .033 -.441 -.019

Cselfeff -.363 .129 -2.818 .006 -.619 -.106

Int_1 -.031 .195 -.157 .876 -.419 .358

Int_2 .081 .201 .402 .689 -.320 .482

Product terms key:

Int_1 : X1 x Cselfeff

Int_2 : X2 x Cselfeff

Test(s) of highest order unconditional interaction(s):

R2-chng F df1 df2 p

X*W .003 .147 2.000 71.000 .863

----------

Focal predict: ExpCond (X)

Mod var: Cselfeff (W)

Data for visualizing the conditional effect of the focal predictor:

Paste text below into a SPSS syntax window and execute to produce plot.

DATA LIST FREE/

ExpCond Cselfeff Bott_Att .

BEGIN DATA.

1.000 -.993 3.610

2.000 -.993 3.279

3.000 -.993 3.728

1.000 .000 3.250

2.000 .000 2.888

3.000 .000 3.448

1.000 .993 2.890

2.000 .993 2.498

3.000 .993 3.169

END DATA.

GRAPH/SCATTERPLOT=

Cselfeff WITH Bott_Att BY ExpCond .

**************************************************************************

OUTCOME VARIABLE:

Brst_Int

Model Summary

R R-sq MSE F df1 df2 p

.881 .776 .362 26.495 9.000 69.000 .000

Model

coeff se t p LLCI ULCI

constant .981 .793 1.237 .220 -.601 2.563

X1 .364 .248 1.466 .147 -.131 .859

X2 -.145 .206 -.703 .484 -.557 .267

MsgEmts .121 .113 1.070 .288 -.104 .346

InfoAcpt -.013 .103 -.126 .900 -.218 .192

Brst_Att .855 .139 6.129 .000 .576 1.133

Bott_Att -.269 .106 -2.548 .013 -.479 -.058

Cselfeff .350 .128 2.746 .008 .096 .605

Int_1 .012 .167 .074 .941 -.322 .346

Int_2 -.113 .173 -.654 .515 -.459 .233

Product terms key:

Int_1 : X1 x Cselfeff

Int_2 : X2 x Cselfeff

Test(s) of highest order unconditional interaction(s):

R2-chng F df1 df2 p

X*W .002 .291 2.000 69.000 .748

----------

Focal predict: ExpCond (X)

Mod var: Cselfeff (W)

Data for visualizing the conditional effect of the focal predictor:

Paste text below into a SPSS syntax window and execute to produce plot.

DATA LIST FREE/

ExpCond Cselfeff Brst_Int .

BEGIN DATA.

1.000 -.993 3.475

2.000 -.993 3.826

3.000 -.993 3.442

1.000 .000 3.822

2.000 .000 4.186

3.000 .000 3.677

1.000 .993 4.170

2.000 .993 4.546

3.000 .993 3.912

END DATA.

GRAPH/SCATTERPLOT=

Cselfeff WITH Brst_Int BY ExpCond .

****************** DIRECT AND INDIRECT EFFECTS OF X ON Y *****************

Relative conditional direct effects of X on Y

Cselfeff Effect se t p LLCI ULCI

X1 -.993 .352 .292 1.206 .232 -.230 .933

X1 .000 .364 .248 1.466 .147 -.131 .859

X1 .993 .376 .305 1.231 .222 -.233 .986

X2 -.993 -.033 .255 -.128 .898 -.541 .475

X2 .000 -.145 .206 -.703 .484 -.557 .267

X2 .993 -.258 .282 -.913 .364 -.821 .305

Relative conditional indirect effects of X on Y:

INDIRECT EFFECT:

ExpCond -> MsgEmts -> Brst_Int

Cselfeff Effect BootSE BootLLCI BootULCI

X1 -.993 -.200 .210 -.635 .212

X1 .000 -.199 .206 -.620 .215

X1 .993 -.198 .207 -.630 .208

Index of moderated mediation:

Index BootSE BootLLCI BootULCI

Cselfeff .001 .032 -.070 .066

Cselfeff Effect BootSE BootLLCI BootULCI

X2 -.993 .012 .075 -.136 .183

X2 .000 .055 .071 -.069 .221

X2 .993 .098 .109 -.107 .337

Index of moderated mediation:

Index BootSE BootLLCI BootULCI

Cselfeff .043 .062 -.047 .195

INDIRECT EFFECT:

ExpCond -> InfoAcpt -> Brst_Int

Cselfeff Effect BootSE BootLLCI BootULCI

X1 -.993 -.004 .056 -.148 .090

X1 .000 -.006 .057 -.136 .105

X1 .993 -.008 .069 -.154 .135

Index of moderated mediation:

Index BootSE BootLLCI BootULCI

Cselfeff -.002 .026 -.048 .067

Cselfeff Effect BootSE BootLLCI BootULCI

X2 -.993 -.011 .087 -.180 .175

X2 .000 -.015 .110 -.212 .233

X2 .993 -.018 .137 -.251 .295

Index of moderated mediation:

Index BootSE BootLLCI BootULCI

Cselfeff -.004 .032 -.058 .074

INDIRECT EFFECT:

ExpCond -> Brst_Att -> Brst_Int

Cselfeff Effect BootSE BootLLCI BootULCI

X1 -.993 .072 .289 -.505 .659

X1 .000 .014 .220 -.478 .404

X1 .993 -.044 .252 -.632 .385

Index of moderated mediation:

Index BootSE BootLLCI BootULCI

Cselfeff -.058 .161 -.429 .219

Cselfeff Effect BootSE BootLLCI BootULCI

X2 -.993 -.220 .262 -.677 .375

X2 .000 -.334 .180 -.675 .033

X2 .993 -.447 .204 -.863 -.055

Index of moderated mediation:

Index BootSE BootLLCI BootULCI

Cselfeff -.114 .152 -.451 .153

INDIRECT EFFECT:

ExpCond -> Bott_Att -> Brst_Int

Cselfeff Effect BootSE BootLLCI BootULCI

X1 -.993 .089 .094 -.073 .304

X1 .000 .097 .089 -.041 .307

X1 .993 .105 .109 -.050 .379

Index of moderated mediation:

Index BootSE BootLLCI BootULCI

Cselfeff .008 .050 -.074 .132

Cselfeff Effect BootSE BootLLCI BootULCI

X2 -.993 -.032 .101 -.261 .171

X2 .000 -.053 .077 -.221 .091

X2 .993 -.075 .099 -.281 .130

Index of moderated mediation:

Index BootSE BootLLCI BootULCI

Cselfeff -.022 .065 -.154 .122

INDIRECT EFFECT:

ExpCond -> MsgEmts -> InfoAcpt -> Brst_Int

Cselfeff Effect BootSE BootLLCI BootULCI

X1 -.993 .008 .065 -.120 .140

X1 .000 .008 .064 -.117 .144

X1 .993 .008 .063 -.117 .145

Index of moderated mediation:

Index BootSE BootLLCI BootULCI

Cselfeff .000 .007 -.014 .016

Cselfeff Effect BootSE BootLLCI BootULCI

X2 -.993 -.001 .015 -.037 .029

X2 .000 -.002 .021 -.049 .038

X2 .993 -.004 .033 -.073 .063

Index of moderated mediation:

Index BootSE BootLLCI BootULCI

Cselfeff -.002 .015 -.035 .029

INDIRECT EFFECT:

ExpCond -> MsgEmts -> Brst_Att -> Brst_Int

Cselfeff Effect BootSE BootLLCI BootULCI

X1 -.993 -.143 .144 -.409 .174

X1 .000 -.142 .140 -.390 .177

X1 .993 -.142 .140 -.394 .174

Index of moderated mediation:

Index BootSE BootLLCI BootULCI

Cselfeff .001 .021 -.042 .049

Cselfeff Effect BootSE BootLLCI BootULCI

X2 -.993 .009 .043 -.104 .077

X2 .000 .040 .046 -.065 .126

X2 .993 .070 .073 -.089 .210

Index of moderated mediation:

Index BootSE BootLLCI BootULCI

Cselfeff .031 .040 -.027 .127

INDIRECT EFFECT:

ExpCond -> MsgEmts -> Bott_Att -> Brst_Int

Cselfeff Effect BootSE BootLLCI BootULCI

X1 -.993 -.057 .072 -.214 .079

X1 .000 -.057 .073 -.221 .076

X1 .993 -.057 .075 -.234 .073

Index of moderated mediation:

Index BootSE BootLLCI BootULCI

Cselfeff .000 .010 -.026 .017

Cselfeff Effect BootSE BootLLCI BootULCI

X2 -.993 .004 .022 -.038 .057

X2 .000 .016 .024 -.024 .076

X2 .993 .028 .037 -.038 .111

Index of moderated mediation:

Index BootSE BootLLCI BootULCI

Cselfeff .012 .019 -.016 .058

INDIRECT EFFECT:

ExpCond -> InfoAcpt -> Brst_Att -> Brst_Int

Cselfeff Effect BootSE BootLLCI BootULCI

X1 -.993 .102 .131 -.167 .381

X1 .000 .142 .112 -.087 .371

X1 .993 .181 .132 -.063 .463

Index of moderated mediation:

Index BootSE BootLLCI BootULCI

Cselfeff .039 .070 -.102 .184

Cselfeff Effect BootSE BootLLCI BootULCI

X2 -.993 .260 .126 .066 .549

X2 .000 .343 .143 .102 .647

X2 .993 .425 .182 .125 .816

Index of moderated mediation:

Index BootSE BootLLCI BootULCI

Cselfeff .083 .064 -.009 .241

INDIRECT EFFECT:

ExpCond -> InfoAcpt -> Bott_Att -> Brst_Int

Cselfeff Effect BootSE BootLLCI BootULCI

X1 -.993 .021 .033 -.034 .101

X1 .000 .029 .031 -.016 .105

X1 .993 .037 .039 -.013 .136

Index of moderated mediation:

Index BootSE BootLLCI BootULCI

Cselfeff .008 .018 -.024 .052

Cselfeff Effect BootSE BootLLCI BootULCI

X2 -.993 .053 .043 .002 .166

X2 .000 .070 .049 .003 .188

X2 .993 .087 .058 .004 .227

Index of moderated mediation:

Index BootSE BootLLCI BootULCI

Cselfeff .017 .016 -.003 .056

INDIRECT EFFECT:

ExpCond -> MsgEmts -> InfoAcpt -> Brst_Att -> Brst_Int

Cselfeff Effect BootSE BootLLCI BootULCI

X1 -.993 -.189 .108 -.451 -.025

X1 .000 -.188 .103 -.422 -.028

X1 .993 -.188 .102 -.421 -.028

Index of moderated mediation:

Index BootSE BootLLCI BootULCI

Cselfeff .001 .022 -.042 .052

Cselfeff Effect BootSE BootLLCI BootULCI

X2 -.993 .012 .048 -.072 .126

X2 .000 .052 .042 .001 .161

X2 .993 .093 .058 .012 .233

Index of moderated mediation:

Index BootSE BootLLCI BootULCI

Cselfeff .041 .032 -.005 .119

INDIRECT EFFECT:

ExpCond -> MsgEmts -> InfoAcpt -> Bott_Att -> Brst_Int

Cselfeff Effect BootSE BootLLCI BootULCI

X1 -.993 -.039 .033 -.124 .000

X1 .000 -.038 .032 -.121 .000

X1 .993 -.038 .031 -.120 .000

Index of moderated mediation:

Index BootSE BootLLCI BootULCI

Cselfeff .000 .005 -.010 .012

Cselfeff Effect BootSE BootLLCI BootULCI

X2 -.993 .002 .011 -.016 .030

X2 .000 .011 .011 .000 .041

X2 .993 .019 .017 .000 .062

Index of moderated mediation:

Index BootSE BootLLCI BootULCI

Cselfeff .008 .009 -.002 .030

*********************** ANALYSIS NOTES AND ERRORS ************************

Level of confidence for all confidence intervals in output:

95.0000

Number of bootstrap samples for percentile bootstrap confidence intervals:

5000

W values in conditional tables are the mean and +/- SD from the mean.

------ END MATRIX -----

Run MATRIX procedure:

**************** PROCESS Procedure for SPSS Version 4.3.1 ****************

Written by Andrew F. Hayes, Ph.D. www.afhayes.com

Documentation available in Hayes (2022), www.guilford.com/p/hayes3

**************************************************************************

Model : CUSTOM

Y : Bott_Int

X : ExpCond

M1 : MsgEmts

M2 : InfoAcpt

M3 : Brst_Att

M4 : Bott_Att

W : Cselfeff

Sample

Size: 79

Coding of categorical X variable for analysis:

ExpCond X1 X2

1.000 .000 .000

2.000 1.000 .000

3.000 .000 1.000

**************************************************************************

OUTCOME VARIABLE:

MsgEmts

Model Summary

R R-sq MSE F df1 df2 p

.829 .687 .439 32.020 5.000 73.000 .000

Model

coeff se t p LLCI ULCI

constant 3.872 .125 30.897 .000 3.622 4.122

X1 -1.650 .183 -9.009 .000 -2.015 -1.285

X2 .459 .181 2.535 .013 .098 .819

Cselfeff .093 .121 .768 .445 -.148 .334

Int_1 .007 .184 .039 .969 -.359 .373

Int_2 .358 .183 1.956 .054 -.007 .724

Product terms key:

Int_1 : X1 x Cselfeff

Int_2 : X2 x Cselfeff

Test(s) of highest order unconditional interaction(s):

R2-chng F df1 df2 p

X*W .020 2.319 2.000 73.000 .106

----------

Focal predict: ExpCond (X)

Mod var: Cselfeff (W)

Data for visualizing the conditional effect of the focal predictor:

Paste text below into a SPSS syntax window and execute to produce plot.

DATA LIST FREE/

ExpCond Cselfeff MsgEmts .

BEGIN DATA.

1.000 -.993 3.780

2.000 -.993 2.123

3.000 -.993 3.883

1.000 .000 3.872

2.000 .000 2.222

3.000 .000 4.331

1.000 .993 3.964

2.000 .993 2.321

3.000 .993 4.779

END DATA.

GRAPH/SCATTERPLOT=

Cselfeff WITH MsgEmts BY ExpCond .

**************************************************************************

OUTCOME VARIABLE:

InfoAcpt

Model Summary

R R-sq MSE F df1 df2 p

.708 .501 .613 12.035 6.000 72.000 .000

Model

coeff se t p LLCI ULCI

constant 1.543 .555 2.779 .007 .436 2.650

X1 .467 .314 1.485 .142 -.160 1.093

X2 1.130 .223 5.069 .000 .685 1.574

MsgEmts .376 .138 2.723 .008 .101 .652

Cselfeff -.035 .143 -.244 .808 -.321 .251

Int_1 .130 .217 .600 .550 -.302 .562

Int_2 .275 .222 1.240 .219 -.167 .718

Product terms key:

Int_1 : X1 x Cselfeff

Int_2 : X2 x Cselfeff

Test(s) of highest order unconditional interaction(s):

R2-chng F df1 df2 p

X*W .011 .772 2.000 72.000 .466

----------

Focal predict: ExpCond (X)

Mod var: Cselfeff (W)

Data for visualizing the conditional effect of the focal predictor:

Paste text below into a SPSS syntax window and execute to produce plot.

DATA LIST FREE/

ExpCond Cselfeff InfoAcpt .

BEGIN DATA.

1.000 -.993 2.899

2.000 -.993 3.236

3.000 -.993 3.755

1.000 .000 2.864

2.000 .000 3.331

3.000 .000 3.994

1.000 .993 2.829

2.000 .993 3.425

3.000 .993 4.232

END DATA.

GRAPH/SCATTERPLOT=

Cselfeff WITH InfoAcpt BY ExpCond .

**************************************************************************

OUTCOME VARIABLE:

Brst_Att

Model Summary

R R-sq MSE F df1 df2 p

.754 .568 .282 13.348 7.000 71.000 .000

Model

coeff se t p LLCI ULCI

constant 2.457 .397 6.195 .000 1.666 3.248

X1 .017 .217 .077 .939 -.415 .449

X2 -.391 .176 -2.217 .030 -.742 -.039

MsgEmts .101 .099 1.024 .309 -.096 .297

InfoAcpt .355 .080 4.435 .000 .195 .514

Cselfeff .450 .097 4.629 .000 .256 .645

Int_1 -.068 .147 -.462 .645 -.362 .226

Int_2 -.134 .152 -.879 .383 -.437 .170

Product terms key:

Int_1 : X1 x Cselfeff

Int_2 : X2 x Cselfeff

Test(s) of highest order unconditional interaction(s):

R2-chng F df1 df2 p

X*W .005 .390 2.000 71.000 .678

----------

Focal predict: ExpCond (X)

Mod var: Cselfeff (W)

Data for visualizing the conditional effect of the focal predictor:

Paste text below into a SPSS syntax window and execute to produce plot.

DATA LIST FREE/

ExpCond Cselfeff Brst_Att .

BEGIN DATA.

1.000 -.993 3.565

2.000 -.993 3.650

3.000 -.993 3.308

1.000 .000 4.013

2.000 .000 4.029

3.000 .000 3.622

1.000 .993 4.460

2.000 .993 4.409

3.000 .993 3.936

END DATA.

GRAPH/SCATTERPLOT=

Cselfeff WITH Brst_Att BY ExpCond .

**************************************************************************

OUTCOME VARIABLE:

Bott_Att

Model Summary

R R-sq MSE F df1 df2 p

.575 .330 .493 5.004 7.000 71.000 .000

Model

coeff se t p LLCI ULCI

constant 4.479 .524 8.545 .000 3.434 5.524

X1 -.361 .286 -1.262 .211 -.932 .209

X2 .199 .233 .853 .397 -.266 .663

MsgEmts -.128 .130 -.984 .329 -.388 .132

InfoAcpt -.230 .106 -2.177 .033 -.441 -.019

Cselfeff -.363 .129 -2.818 .006 -.619 -.106

Int_1 -.031 .195 -.157 .876 -.419 .358

Int_2 .081 .201 .402 .689 -.320 .482

Product terms key:

Int_1 : X1 x Cselfeff

Int_2 : X2 x Cselfeff

Test(s) of highest order unconditional interaction(s):

R2-chng F df1 df2 p

X*W .003 .147 2.000 71.000 .863

----------

Focal predict: ExpCond (X)

Mod var: Cselfeff (W)

Data for visualizing the conditional effect of the focal predictor:

Paste text below into a SPSS syntax window and execute to produce plot.

DATA LIST FREE/

ExpCond Cselfeff Bott_Att .

BEGIN DATA.

1.000 -.993 3.610

2.000 -.993 3.279

3.000 -.993 3.728

1.000 .000 3.250

2.000 .000 2.888

3.000 .000 3.448

1.000 .993 2.890

2.000 .993 2.498

3.000 .993 3.169

END DATA.

GRAPH/SCATTERPLOT=

Cselfeff WITH Bott_Att BY ExpCond .

**************************************************************************

OUTCOME VARIABLE:

Bott_Int

Model Summary

R R-sq MSE F df1 df2 p

.845 .715 .438 19.190 9.000 69.000 .000

Model

coeff se t p LLCI ULCI

constant 3.901 .872 4.472 .000 2.161 5.641

X1 -.266 .273 -.975 .333 -.811 .278

X2 .112 .227 .493 .624 -.341 .565

MsgEmts -.107 .124 -.862 .391 -.355 .141

InfoAcpt -.036 .113 -.321 .749 -.262 .190

Brst_Att -.572 .153 -3.730 .000 -.878 -.266

Bott_Att .397 .116 3.423 .001 .166 .629

Cselfeff -.221 .140 -1.572 .121 -.500 .059

Int_1 -.384 .184 -2.087 .041 -.751 -.017

Int_2 -.007 .191 -.035 .972 -.387 .374

Product terms key:

Int_1 : X1 x Cselfeff

Int_2 : X2 x Cselfeff

Test(s) of highest order unconditional interaction(s):

R2-chng F df1 df2 p

X*W .022 2.637 2.000 69.000 .079

----------

Focal predict: ExpCond (X)

Mod var: Cselfeff (W)

Conditional effects of the focal predictor at values of the moderator(s):

(These are also the relative conditional direct effects of X on Y)

Moderator value(s):

Cselfeff -.993

Effect se t p LLCI ULCI

X1 .115 .321 .359 .721 -.525 .755

X2 .119 .280 .423 .673 -.440 .677

Test of equality of conditional means

F df1 df2 p

.115 2.000 69.000 .891

Estimated conditional means being compared:

ExpCond Bott_Int

1.000 2.670

2.000 2.785

3.000 2.788

----------

Moderator value(s):

Cselfeff .000

Effect se t p LLCI ULCI

X1 -.266 .273 -.975 .333 -.811 .278

X2 .112 .227 .493 .624 -.341 .565

Test of equality of conditional means

F df1 df2 p

.694 2.000 69.000 .503

Estimated conditional means being compared:

ExpCond Bott_Int

1.000 2.451

2.000 2.185

3.000 2.563

----------

Moderator value(s):

Cselfeff .993

Effect se t p LLCI ULCI

X1 -.648 .336 -1.927 .058 -1.318 .023

X2 .105 .310 .339 .736 -.514 .724

Test of equality of conditional means

F df1 df2 p

2.107 2.000 69.000 .129

Estimated conditional means being compared:

ExpCond Bott_Int

1.000 2.232

2.000 1.584

3.000 2.337

Data for visualizing the conditional effect of the focal predictor:

Paste text below into a SPSS syntax window and execute to produce plot.

DATA LIST FREE/

ExpCond Cselfeff Bott_Int .

BEGIN DATA.

1.000 -.993 2.670

2.000 -.993 2.785

3.000 -.993 2.788

1.000 .000 2.451

2.000 .000 2.185

3.000 .000 2.563

1.000 .993 2.232

2.000 .993 1.584

3.000 .993 2.337

END DATA.

GRAPH/SCATTERPLOT=

Cselfeff WITH Bott_Int BY ExpCond .

****************** DIRECT AND INDIRECT EFFECTS OF X ON Y *****************

Relative conditional direct effects of X on Y

Cselfeff Effect se t p LLCI ULCI

X1 -.993 .115 .321 .359 .721 -.525 .755

X1 .000 -.266 .273 -.975 .333 -.811 .278

X1 .993 -.648 .336 -1.927 .058 -1.318 .023

X2 -.993 .119 .280 .423 .673 -.440 .677

X2 .000 .112 .227 .493 .624 -.341 .565

X2 .993 .105 .310 .339 .736 -.514 .724

Relative conditional indirect effects of X on Y:

INDIRECT EFFECT:

ExpCond -> MsgEmts -> Bott_Int

Cselfeff Effect BootSE BootLLCI BootULCI

X1 -.993 .177 .218 -.242 .623

X1 .000 .177 .215 -.237 .613

X1 .993 .176 .215 -.238 .617

Index of moderated mediation:

Index BootSE BootLLCI BootULCI

Cselfeff -.001 .029 -.060 .060

Cselfeff Effect BootSE BootLLCI BootULCI

X2 -.993 -.011 .067 -.189 .100

X2 .000 -.049 .075 -.244 .061

X2 .993 -.087 .115 -.347 .106

Index of moderated mediation:

Index BootSE BootLLCI BootULCI

Cselfeff -.038 .057 -.173 .059

INDIRECT EFFECT:

ExpCond -> InfoAcpt -> Bott_Int

Cselfeff Effect BootSE BootLLCI BootULCI

X1 -.993 -.012 .069 -.179 .123

X1 .000 -.017 .071 -.175 .126

X1 .993 -.022 .085 -.209 .149

Index of moderated mediation:

Index BootSE BootLLCI BootULCI

Cselfeff -.005 .032 -.077 .060

Cselfeff Effect BootSE BootLLCI BootULCI

X2 -.993 -.031 .108 -.270 .170

X2 .000 -.041 .136 -.330 .209

X2 .993 -.051 .168 -.400 .256

Index of moderated mediation:

Index BootSE BootLLCI BootULCI

Cselfeff -.010 .038 -.096 .064

INDIRECT EFFECT:

ExpCond -> Brst_Att -> Bott_Int

Cselfeff Effect BootSE BootLLCI BootULCI

X1 -.993 -.048 .200 -.491 .336

X1 .000 -.010 .149 -.304 .307

X1 .993 .029 .172 -.289 .414

Index of moderated mediation:

Index BootSE BootLLCI BootULCI

Cselfeff .039 .113 -.151 .313

Cselfeff Effect BootSE BootLLCI BootULCI

X2 -.993 .148 .178 -.248 .479

X2 .000 .224 .134 -.027 .492

X2 .993 .300 .160 .030 .647

Index of moderated mediation:

Index BootSE BootLLCI BootULCI

Cselfeff .077 .104 -.090 .326

INDIRECT EFFECT:

ExpCond -> Bott_Att -> Bott_Int

Cselfeff Effect BootSE BootLLCI BootULCI

X1 -.993 -.132 .133 -.427 .116

X1 .000 -.144 .116 -.388 .071

X1 .993 -.156 .137 -.455 .085

Index of moderated mediation:

Index BootSE BootLLCI BootULCI

Cselfeff -.012 .069 -.155 .123

Cselfeff Effect BootSE BootLLCI BootULCI

X2 -.993 .047 .141 -.258 .319

X2 .000 .079 .106 -.147 .287

X2 .993 .111 .142 -.169 .400

Index of moderated mediation:

Index BootSE BootLLCI BootULCI

Cselfeff .032 .094 -.148 .233

INDIRECT EFFECT:

ExpCond -> MsgEmts -> InfoAcpt -> Bott_Int

Cselfeff Effect BootSE BootLLCI BootULCI

X1 -.993 .023 .082 -.139 .197

X1 .000 .023 .080 -.139 .188

X1 .993 .022 .079 -.142 .183

Index of moderated mediation:

Index BootSE BootLLCI BootULCI

Cselfeff .000 .009 -.023 .016

Cselfeff Effect BootSE BootLLCI BootULCI

X2 -.993 -.001 .020 -.049 .037

X2 .000 -.006 .026 -.062 .048

X2 .993 -.011 .040 -.095 .076

Index of moderated mediation:

Index BootSE BootLLCI BootULCI

Cselfeff -.005 .019 -.045 .036

INDIRECT EFFECT:

ExpCond -> MsgEmts -> Brst_Att -> Bott_Int

Cselfeff Effect BootSE BootLLCI BootULCI

X1 -.993 .096 .103 -.112 .315

X1 .000 .095 .102 -.108 .311

X1 .993 .095 .104 -.107 .311

Index of moderated mediation:

Index BootSE BootLLCI BootULCI

Cselfeff .000 .015 -.031 .033

Cselfeff Effect BootSE BootLLCI BootULCI

X2 -.993 -.006 .030 -.057 .076

X2 .000 -.026 .032 -.089 .044

X2 .993 -.047 .052 -.157 .056

Index of moderated mediation:

Index BootSE BootLLCI BootULCI

Cselfeff -.021 .029 -.096 .016

INDIRECT EFFECT:

ExpCond -> MsgEmts -> Bott_Att -> Bott_Int

Cselfeff Effect BootSE BootLLCI BootULCI

X1 -.993 .084 .099 -.117 .279

X1 .000 .084 .099 -.112 .288

X1 .993 .084 .100 -.111 .302

Index of moderated mediation:

Index BootSE BootLLCI BootULCI

Cselfeff .000 .014 -.026 .035

Cselfeff Effect BootSE BootLLCI BootULCI

X2 -.993 -.005 .032 -.069 .064

X2 .000 -.023 .033 -.100 .036

X2 .993 -.041 .054 -.163 .058

Index of moderated mediation:

Index BootSE BootLLCI BootULCI

Cselfeff -.018 .030 -.094 .025

INDIRECT EFFECT:

ExpCond -> InfoAcpt -> Brst_Att -> Bott_Int

Cselfeff Effect BootSE BootLLCI BootULCI

X1 -.993 -.069 .090 -.265 .102

X1 .000 -.095 .076 -.256 .050

X1 .993 -.121 .090 -.312 .043

Index of moderated mediation:

Index BootSE BootLLCI BootULCI

Cselfeff -.026 .049 -.126 .074

Cselfeff Effect BootSE BootLLCI BootULCI

X2 -.993 -.174 .092 -.385 -.032

X2 .000 -.229 .107 -.462 -.050

X2 .993 -.285 .135 -.580 -.061

Index of moderated mediation:

Index BootSE BootLLCI BootULCI

Cselfeff -.056 .044 -.160 .007

INDIRECT EFFECT:

ExpCond -> InfoAcpt -> Bott_Att -> Bott_Int

Cselfeff Effect BootSE BootLLCI BootULCI

X1 -.993 -.031 .045 -.138 .048

X1 .000 -.043 .041 -.136 .023

X1 .993 -.055 .050 -.171 .021

Index of moderated mediation:

Index BootSE BootLLCI BootULCI

Cselfeff -.012 .025 -.065 .035

Cselfeff Effect BootSE BootLLCI BootULCI

X2 -.993 -.078 .051 -.202 -.003

X2 .000 -.103 .059 -.235 -.005

X2 .993 -.128 .074 -.292 -.006

Index of moderated mediation:

Index BootSE BootLLCI BootULCI

Cselfeff -.025 .023 -.083 .006

INDIRECT EFFECT:

ExpCond -> MsgEmts -> InfoAcpt -> Brst_Att -> Bott_Int

Cselfeff Effect BootSE BootLLCI BootULCI

X1 -.993 .127 .076 .013 .312

X1 .000 .126 .074 .013 .303

X1 .993 .126 .075 .012 .299

Index of moderated mediation:

Index BootSE BootLLCI BootULCI

Cselfeff -.001 .015 -.032 .032

Cselfeff Effect BootSE BootLLCI BootULCI

X2 -.993 -.008 .032 -.085 .048

X2 .000 -.035 .029 -.107 .000

X2 .993 -.062 .039 -.157 -.006

Index of moderated mediation:

Index BootSE BootLLCI BootULCI

Cselfeff -.027 .022 -.077 .005

INDIRECT EFFECT:

ExpCond -> MsgEmts -> InfoAcpt -> Bott_Att -> Bott_Int

Cselfeff Effect BootSE BootLLCI BootULCI

X1 -.993 .057 .041 .000 .158

X1 .000 .057 .039 .000 .152

X1 .993 .057 .038 .000 .146

Index of moderated mediation:

Index BootSE BootLLCI BootULCI

Cselfeff .000 .007 -.017 .013

Cselfeff Effect BootSE BootLLCI BootULCI

X2 -.993 -.004 .015 -.037 .029

X2 .000 -.016 .014 -.053 .001

X2 .993 -.028 .022 -.086 .000

Index of moderated mediation:

Index BootSE BootLLCI BootULCI

Cselfeff -.012 .013 -.048 .002

*********************** ANALYSIS NOTES AND ERRORS ************************

Level of confidence for all confidence intervals in output:

95.0000

Number of bootstrap samples for percentile bootstrap confidence intervals:

5000

W values in conditional tables are the mean and +/- SD from the mean.

------ END MATRIX -----
